# Risk of incident tuberculosis after severe COVID-19: a nationwide cohort study

**DOI:** 10.1038/s41467-026-74528-5

**Published:** 2026-06-29

**Authors:** Salvador Vargas-García, Lourdes García-García, Christian García, Natalia Vergara, Nadia Escobar, Luis A. Villarroel, Kasim Allel, Eduardo A. Undurraga, María Elvira Balcells

**Affiliations:** 1 School of Public Health, Pontificia Universidad Católica de Chile, Santiago, Chile; 2 Instituto de Ciencias Biomédicas, Universidad Autónoma de Ciudad Juárez, Ciudad Juárez, Chihuahua, México; 3 SENTINET: Surveillance, Epidemiology, and New Technologies for Infectious Emerging Threats, Santiago, Chile; 4 Centro de Investigación sobre Enfermedades Infecciosas (CISEI), Instituto Nacional de Salud Pública, Cuernavaca, México; 5 Programa Centro de Salud Pública, Universidad de, Santiago, Chile; 6 Servicio de Salud Metropolitano Oriente, Santiago, Chile; 7 Department of Epidemiology, Ministry of Health, Santiago, Chile; 8 National Tuberculosis Control and Elimination Program, Ministry of Health, Santiago, Chile; 9 Nuffield Department of Primary Care Health Sciences, University of Oxford, Oxford, UK; 10 Department of Infectious Diseases, School of Medicine, Pontificia Universidad Católica de Chile, Santiago, Chile; 11 Escuela de Gobierno, Pontificia Universidad Católica de Chile, Santiago, Chile

**Keywords:** Tuberculosis, SARS-CoV-2

## Abstract

Severe viral infections can disrupt host immunity and increase susceptibility to secondary pathogens. Experimental and preclinical studies suggest that severe viral pneumonia may also trigger reactivation of *Mycobacterium tuberculosis* infection, yet population-level evidence remains limited. We conducted a nationwide retrospective cohort study in Chile, including more than 3.6 million adults with confirmed SARS-CoV-2 infection, to assess the risk of incident tuberculosis during follow-up. Individuals who developed severe COVID-19 requiring hospitalization had more than an eightfold higher hazard of tuberculosis within one year compared with those with non-severe disease. SARS-CoV-2 vaccination modified this association: the excess risk was substantially greater among unvaccinated individuals, whereas prior vaccination attenuated it. These findings indicate that severe COVID-19 is associated with an increased short- and long-term risk of tuberculosis and support the integration of targeted tuberculosis screening and preventive strategies into post-COVID-19 care, particularly among patients with severe disease.

## Abstract (web summary)

In their retrospective cohort study, authors show that severe COVID-19 is associated with an eightfold higher tuberculosis risk in a nationwide cohort of 3.6 million adults. Risk peaks in the first six months and declines over time. COVID-19 vaccination substantially reduces this risk.

## Introduction

In 2024, an estimated 10.7 million individuals developed tuberculosis (TB) globally, with approximately 1.2 million deaths, making TB once again the leading cause of death from a single infectious agent^1^. The burden continues to fall disproportionately on low- and middle-income countries, where under-resourced health systems impede progress toward global elimination targets^2–4^. The COVID-19 pandemic further disrupted TB control efforts, contributing to an estimated half a million excess TB deaths between 2020 and 2022 and reversing over a decade of gains in life expectancy^5^.

Before widespread vaccine availability, approximately 14% of SARS-CoV-2 infections progressed to severe disease requiring hospitalization, and around 5% to critical illness, including acute respiratory distress syndrome, multi-organ failure, and death^6^. Severe COVID-19 is characterized by an intense hyperinflammatory response, with elevated cytokines such as IL-6 and TNF-α and extensive pulmonary inflammation^7^. This dysregulated immune activation may persist beyond the acute phase, leading to T-cell dysfunction and transient immunosuppression^8^, thereby increasing susceptibility to secondary bacterial and fungal infections, as well as to long-term sequelae, including neurocognitive and cardiovascular complications^9,10^.

Emerging evidence, mostly from high-TB incidence countries, suggests that individuals recovering from COVID-19 may be at increased risk of TB development^11,12^. Similar associations have been described following other respiratory viral infections, including SARS-CoV, MERS-CoV, influenza, and measles^13,14^. In addition, immunomodulatory therapies used to treat severe COVID-19, such as corticosteroids and interleukin-6 (IL-6) inhibitors (e.g., tocilizumab), while effective in reducing mortality, may further increase susceptibility to secondary infections and *M. tuberculosis* (TBI) reactivation^15,16^.

Despite these observations, important uncertainties remain. The magnitude of TB risk following SARS-CoV-2 infection has not been well defined at the population level. It is unclear whether any excess risk is limited to severe COVID-19 pneumonia cases, potentially driven by immune dysregulation or immunosuppressive therapy, or whether it also extends to those with mild or moderate illness. The duration of any elevated TB risk after SARS-CoV-2 infection is likewise unknown. Given the high global prevalence of TBI^17^, clarifying these associations is critical to inform targeted screening, preventive, and therapeutic strategies. Here, we analyze the association between COVID-19 severity and the subsequent risk of new-onset TB within one year of SARS-CoV-2 diagnosis in a nationwide cohort in Chile. We show that severe COVID-19 is associated with substantially increased short and long-term risks of TB, particularly during the first six months of follow-up, and that prior SARS-CoV-2 vaccination attenuates this association. These findings support the integration of targeted TB screening and prevention strategies into post-COVID-19 care, particularly for individuals recovering from severe disease.

## Results

### Study population

The final study cohort included 3,607,175 adults with laboratory-confirmed SARS-CoV-2 infection recorded between March 1, 2020, and October 28, 2022. Of these, 94.16% (*n* = 3,396,533) were non-hospitalized; 2.76% (*n* = 99,717) were hospitalized, and 3.07% (*n* = 110,925) were admitted to the intensive care unit (ICU). The cohort’s median age was 40 years (IQR: 29–55), and 53.15% (*n* = 1,917,280) were female. Additionally, 6.63% (*n* = 239,435) were identified as migrants, 2.33% (*n* = 86,402) had an indigenous status, and 3.97% (*n* = 143,453) were healthcare workers. The majority (*n* = 2,984,697; 82.74%) was affiliated with Chile’s public healthcare insurance system. Pre-existing conditions were present in over half of participants: 5.22% (*n* = 188,467) had diabetes mellitus, 3.16% (*n* = 114,216) had at least one major chronic comorbidity (renal, liver or pulmonary), and 46.05% (*n* = 1,661,032) had other comorbidities, including cardiovascular and unspecified conditions. Only 16.46% (*n* = 593,934) had a complete SARS-CoV-2 vaccination scheme prior to infection. A detailed summary of baseline characteristics is presented in Table 1.

**Table 1:** Characteristics of the SARS-CoV-2 infection national cohort according to new-onset TB within one year of follow-up

Variables / Model sample	New-onset TB within one year of follow-up			
	All individuals	Non-TB ^a^	Active TB ^a^	*p*-value ^*b*^
Participants (*n*)	3,607,175	3,606,442 (99.98%)	733 (0.02%)	
COVID-19 severity (%)				<0.0001
Non-hospitalized	3,396,533 (94.16)	3,396,095 (94.17)	438 (59.75)	
Hospitalized	99,717 (2.76)	99,581 (2.76)	136 (18.55)	
ICU-admitted	110,925 (3.07)	110,766 (3.07)	159 (21.69)	
COVID-19 vaccinated ^c^ (%)	593,934 (16.46)	593,616 (16.45)	318 (43.38)	<0.0001
Diabetes (%)	188,467 (5.22)	188,367 (5.22)	100 (13.64)	<0.0001
Major chronic comorbidities ^d^ (%)	114,216 (3.16)	114,123 (3.16)	93 (12.30)	<0.0001
No illness reported (%)	1,661,032 (46.04)	1,660,787 (46.05)	245 (33.42)	<0.0001
Age, median (IQR)	40 (29–55)	40 (29–55)	50 (33–64)	<0.0001
Sex, Male (%)	1,689,895 (46.84)	1,689,444 (46.84)	451 (61.52)	<0.0001
Migrant ^e^ (%)	239,435 (6.63)	239,306 (6.63)	129 (17.59)	<0.0001
Indigenous status ^f^ (%)	86,402 (2.33)	86,379 (2.39)	23 (3.13)	0.189
Healthcare worker (%)	143,453 (3.97)	143,434 (3.97)	10 (2.59)	0.055
Public healthcare insurance ^g^ (%)	2,984,697 (82.74)	2,964,007 (82.18)	690 (94.13)	<0.0001
Other comorbidities ^h^ (%)	1,661,032 (46.05)	1,660,787 (46.05)	245 (33.42)	<0.0001

Notes. TB: tuberculosis.

^a^ Median (IQR) or frequency (%).

^b^ Pearson´s Chi-squared test; Wilcoxon rank sum test; Fisher´s exact test. All statistical tests were two-sided.

^c^ SARS-CoV-2 vaccination status was considered complete if individuals had received two doses of any SARS-CoV-2 vaccine, regardless of vaccine type or combination (including Pfizer-BioNTech [BNT162b2], Sinovac [CoronaVac], AstraZeneca [ChAdOx1-S], or mixed schedules), or a single dose of the CanSino (Ad5-nCoV) vaccine, prior to the SARS-CoV-2 infection identification.

^d^ At least one major chronic comorbidities (Lung, kidney, or liver disease) registered at the time of SARS-CoV-2 identification.

^e^ Migrant status, regardless of legal residency, as recorded by the Chilean health system.

^f^ Self-reporting of having an indigenous status.

^g^ Belong to the Chilean public healthcare insurance.

^h^ Presence of at least one comorbidity, including cardiovascular disease, neurological conditions, obesity, hypertension, and other non-specified comorbidities.

*ICU* = Intensive care unit. *IQR* = Interquartile range.

### One-year risk of incident TB in the complete cohort

During a total of 3,604,376 person-years of follow-up, 733 individuals (0.02%) developed new-onset TB within one year of SARS-CoV-2 infection. Compared to those who did not develop TB, individuals with new-onset TB were older (median: 50 vs 40 years, *p* < 0.001), more likely to be males (61.52% vs 46.84%, *p* < 0.0001) or migrants (17.59% vs 6.63%, *p* < 0.0001), had a higher prevalence of comorbidities (12.30% vs 3.16%, *p* < 0.0001), and a greater proportion had received SARS-CoV-2 vaccination (43.38% vs 16.45%, *p* < 0.0001) (Table 1). The median time from SARS-CoV-2 diagnosis to new-onset TB was 127 days (IQR: 30–238). Approximately 35% of TB cases occurred within the first two months following SARS-CoV-2 infection (Fig. S1).

Among the 3,607,175 individuals with confirmed SARS-CoV-2 infection, 96.97% (*n* = 3,606,442) remained alive and free of TB disease at the end of the one-year follow-up period. Informative censoring due to non-TB-related deaths occurred in 1.51% (*n* = 54,474) of participants, of which 68.57% (*n* = 37,377) were attributed to COVID-19 and 31.36% (*n* = 17,097) to other causes.

### One-year risk of incident TB by COVID-19 severity

**Fig. 1:**
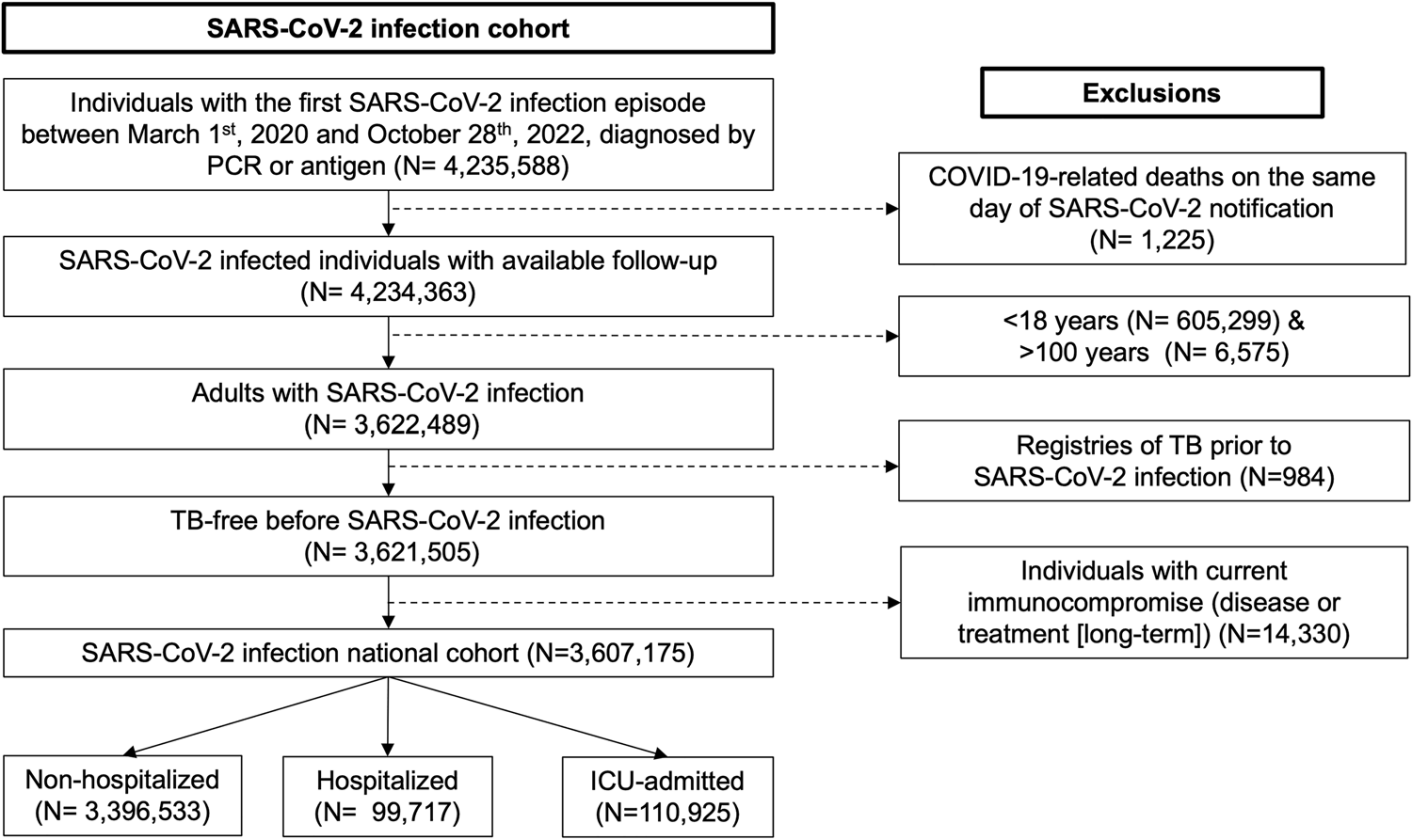
Flow diagram of the national SARS-CoV-2 cohort, study population selection, and eligibility criteria. Cohort diagram of SARS-CoV-2 infections, including study population selection and eligibility criteria. Notes: TB Tuberculosis, COVID-19 Coronavirus Disease 2019, ICU Intensive Care Unit. N Number of observations.

Time-to-event curves showed differences in the cumulative risk of new-onset TB according to COVID-19 severity. Individuals who required COVID-19 hospitalization, with or without ICU admission, exhibited a higher risk of incident TB compared with non-hospitalized individuals (Fig. 1). At one year of follow-up, the crude hazard ratio (cHR) for TB was 10.58 (95% CI: 8.73–12.83) among hospitalized patients and 11.12 (95% CI: 9.27–13.33) among those admitted to ICU, relative to non-hospitalized cases. The excess risk declined over time (ln (p) = −0.44; 95% CI: −0.51–−0.37), indicating a stronger association in the early post-COVID period.

In multivariable models adjusting for age, sex, migrant status, enrollment in the public healthcare system, diabetes mellitus, and other comorbidities, the association between COVID-19 severity and incident TB remained robust. Compared with non-hospitalized individuals, the adjusted hazard ratio (aHR) was 8.92 (95% CI: 7.28–10.94) among hospitalized patients, and 8.31 (95% CI: 6.71–10.28) among ICU-admitted patients (Table 2). The time-dependent decline in TB risk persisted after adjustment (ln (*p*) = −0.44, 95% CI: −0.51−−0.37).

**Table 2:** Factors associated with new-onset TB within one year of follow-up in the SARS-CoV-2 infection national cohort in Chile (2020–2022)

Model sample and independent variables	N/ person-years	TB incidence rate per 100,000 person-years (95% CI)	Crude HR (95% CI)	Adjusted HR (95% CI)
**A. Complete cohort**	733/ 3,604,261	20.34 (18.89–21.86)		
COVID-19 severity				
Non-hospitalized	438/ 3,393,990	12.91 (11.72–14.17)	Ref	Ref
Hospitalized	136/ 99,547.07	136.62 (114.64–161.58)	10.58 (8.73–12.83)	8.92 (7.28–10.94)
ICU-admited	159/ 107,239	148.27 (126.13–173.17)	11.12 (9.27–13.33)	8.31 (6.71–10.28)
Diabetes	100/ 188,271.3	53.11 (43.22–64.42)	2.86 (2.32–3.53)	1.26 (1.00–1.58)
Major chronic comorbidities^b^	93/ 114,075.4	81.53 (65.81–99.26)	4.44 (3.57–5.52)	2.31 (1.84–2.91)
Age (years)	–	–	1.02 (1.01–1.02)	1.00 (0.99–1.00)
Sex (Male)	451/ 1,688,461	26.71 (24.3–29.29)	1.81 (1.56–2.10)	1.74 (1.49–2.02)
Public health insurance ^d^	690/ 2,962,248	23.29 (21.59–25.1)	3.47 (2.55–4.73)	2.91 (2.13–3.97)
Migrant ^c^	129/ 239,189.1	53.93 (45.03–14.05)	3.00 (2.48–3.63)	3.39 (2.79-4.13)
ln (p) ^e^	–	–	–	−0.44 (−0.51– −0.37)
**B. COVID-19 unvaccinated**^**a**^	415/ 3,010,915	13.78 (12.49–15.18)		
COVID-19 severity				
Non-hospitalized	214/ 2,861,190	7.48 (6.51–8.55)	Ref	Ref
Hospitalized	94/ 67,653	138.94 (143.2–170.01)	18.57 (14.57–23.67)	17.77 (13.48-22.41)
ICU-admitted	107/ 82,071.42	130.37 (106.86–157.52)	17.42 (13.81-221.98)	16.16 (12.31-21.22)
Diabetes	59/ 147,175.6	40.09 (30.52–51.71)	3.22 (2.44–4.25)	1.34 (0.99-1.81)
Major chronic comorbidities^b^	54/ 89,195.22	60.54 (45.48–78.99)	4.89 (3.68–6.52)	2.37 (1.75-3.20)
Age (years)	–	–	1.01 (1.01–1.02)	0.99 (0.98-1.00)
Sex (Male)	255/ 1,447,680	17.26 (15.2–19.51)	1.65 (1.36–2.01)	1.59 (1.31-1.95)
Public health insurance ^d^	394/ 2,511,080	15.69 (14.18–17.32)	3.73 (2.40–5.79)	2.37 (1.75-3.20)
Migrant ^c^	94/ 218,392	43.04 (34.78–52.67)	3.74 (2.98–4.71)	4.02 (3.17-5-11)
ln (p) ^e^	–	–	–	−0.54 (−0.63- −0.44)
**C. COVID-19 vaccinated**^**a**^	318/ 593,346.6	53.59 (47.87–59.82)		
COVID-19 severity				
Non-hospitalized	224/ 532,799.6	42.04 (36.72–47.92)	Ref	Ref
Hospitalized	42/ 31,894.05	131.64 (94.92–177.96)	3.13 (2.25–4.35)	2.11 (1.49–2.97)
ICU-admitted	52/ 28,652.48	181.49 (135.57–237.93)	4.31 (3.19–5.83)	2.46 (1.76–3.45)
Diabetes	41/ 41,095.74	99.77 (71.61–135.32)	1.98 (1.43–2.76)	1.12 (0.79–1.59)
Major chronic comorbidities^b^	39/ 24,880.17	156.75 (111.49–214.22)	3.19 (2.28–4.46)	2.19 (1.54–3.12)
Age (years)	–	–	1.01 (1.01–1.02)	1.00 (0.99–1.00)
Sex (Male)	196/ 210,781.2	92.99 (80.43–106.95)	2.91 (2.32–3.65)	2.83 (2.24–3.58)
Public health insurance ^c^	296/ 451,168.4	65.61 (9.70–23.43)	4.25 (2.75–6.53)	4.07 (2.63–6.30)
Migrant ^d^	35/ 20,796.82	168.3 (117.26–233.99)	3.40 (2.39–4.83)	3.89 (2.72–5.56)
ln (p) ^e^	–	–	–	−0.30 (−0.41– −0.19)

Notes: **Model A. Complete Cohort** (including all individuals [*N* = 3,607,175]). **Model B. COVID-19 unvaccinated cohort** (including only those who did not register a complete vaccination scheme prior to SARS-CoV-2 infection identification [*N* = 3,013,241 {83.53%}]). **Model C. COVID-19 vaccinated cohort** (including only those who registered a complete vaccination scheme prior to SARS-CoV-2 infection identification [*N* = 593,934 {16.46%}]).

*TB* Tuberculosis, *95% CI* Confidence Intervals in parentheses. N persons years: events (New-onset TB)/time of follow-up in years, *Ref.* Reference category, *HR* = Hazard ratios, *ICU* = Intensive care units.

^a^ SARS-CoV-2 vaccination status was considered complete if individuals had received two doses of any SARS-CoV-2 vaccine, regardless of vaccine type or combination (including Pfizer-BioNTech [BNT162b2], Sinovac [CoronaVac], AstraZeneca [ChAdOx1-S], or mixed schedules), or a single dose of the CanSino (Ad5-nCoV) vaccine, prior to the SARS-CoV-2 infection identification.

^b^ At least one major chronic comorbidities (Lung, kidney or liver disease) registered at the time of SARS-CoV-2 identification.

^c^ Belong to the Chilean public healthcare insurance.

^d^ Migrant status, regardless of legal residency, as recorded by the Chilean health system.

^e^ The natural logarithm of the Weibull shape parameter. ln = natural logarithm. All statistical tests were two-sided.

Several covariates were independently associated with increased TB risk, including male sex (aHR 1.74, 95% CI: 1.49–2.02), migrant status (aHR 3.39, 95% CI: 2.79–4.13), enrollment in the public healthcare system (aHR 2.91, 95% CI: 2.13–3.97), and the presence of at least one major chronic comorbidity (aHR 2.31, 95% CI: 1.84–2.91). Diabetes was marginally associated with TB risk (aHR 1.26, 95% CI: 1.00–1.58).

Stratified analysis by SARS-CoV-2 vaccination status revealed effect modification. Among unvaccinated individuals, the association between severe COVID-19 and incident TB was markedly stronger (aHR 17.77, 95% CI: 13.48–22.41 for hospitalized patients; aHR 16.16, 95% CI: 12.31–21.22 for ICU-admitted patients). In contrast, among vaccinated individuals, the corresponding estimates were substantially attenuated (aHR 2.11, 95% CI: 1.49–2.97; and aHR 2.46, 95% CI: 1.76–3.45, respectively) (Table 2). Baseline characteristics by vaccination status are shown in Supplementary Table S1, and descriptive characteristics stratified by COVID-19 severity and vaccination status are presented in Table S2.

### Short- and long-term risk of incident TB by time periods

When restricting follow-up to the first six months after SARS-CoV-2 infection, the magnitude of association between COVID-19 severity and incident TB was nearly twice that observed at one year. The aHR was 15.81 (95% CI, 12.37–20.21) among hospitalized individuals and 16.12 (95% CI, 12.46–20.87) among those admitted to the ICU. With longer follow-up (median 340 days, IQR:112-618), the association persisted but was attenuated. Compared with non-hospitalized individuals, the aHR for TB was 4.84 (95% CI: 4.10–5.64) among those having been hospitalized, and 3.46 (95% CI: 2.91–4.11) among those having required ICU-admission (Table 3). The direction and magnitude of associations for other covariates remained consistent across longer follow-up periods.

**Table 3:** Factors associated with new-onset TB during early (six months) and extended (three-years) follow-up after SARS-CoV-2 infection, Chile (2020–2022)

Variables/ Model	Crude HR (CI 95%)	Adjusted HR (CI 95%)
**A. Early follow-up (six months)**		
TB cases (N/ person-years)	448/1,777519.10	–
TB incidence rate per 100,000 person-years	27.45 (25.08–30.00)	–
COVID-19 severity		
Non-hospitalized	Ref	Ref
Hospitalized	17.69 (14.02–22.32)	15.81 (12.37–20.21)
ICU-admited	19.44 (15.63–24.18)	16.12 (12.46–20.87)
Diabetes	3.24 (2.50–4.20)	1.19 (0.90–1.56)
Major chronic comorbidities ^a^	5.28 (4.07–6.86)	2.34 (1.78–3.09)
Age (years)	1.02 (1.01–1.03)	0.99 (0.99–1.00)
Sex (Male)	1.80 (1.49–2.17)	1.66 (1.37–2.01)
Public healthcare insurance ^b^	4.00 (2.63–6.09)	3.16 (2.07–4.81)
Migrant ^c^	3.24 (2.56–4.11)	3.77 (2.94–4.82)
ln (p) ^d^		−0-49 (−0.40–−0.59)
**B. Extended follow-up (three years)**		
TB cases (N/ person-years)	1391/ 7,895,422.71	–
TB incidence rate per 100,000 person-years	17.61 (16.7–18.5)	–
COVID-19 severity		
Non-hospitalized	Ref	Ref
Hospitalized	6.17 (5.30–7.18)	4.84 (4.10–5.64)
ICU-admited	5.28 (4.54–6.15)	3.46 (2.91–4.11)
Diabetes	2.96 (2.55–3.43)	1.63 (1.39–1.92)
Major chronic comorbidities ^a^	3.59 (3.03–4.25)	2.20 (1.84–2.63)
Age (years)	1.02 (1.01–1.02)	1.00 (0.99–1.00)
Sex (Male)	1.64 (1.47–1.83)	1.66 (1.49–1.85)
Public healthcare insurance ^b^	3.45 (2.75–4.33)	3.02 (2.40–3.61)
Migrant ^c^	2.77 (2.41–3.18)	3.13 (2.71–3.61)
ln (p) ^d^		−0-34 (−0.40–−0.29)

*TB* Tuberculosis, *CI* Confidence Interval. N persons years: events (New-onset TB)/time of follow-up in years, *Ref* Reference category, *HR* Hazard ratios, *ICU* Intensive care units.

^a^ At least one major chronic comorbidities (lung, kidney, or liver disease) was reported at the time of SARS-CoV-2 identification.

^b^ Belong to the Chilean public healthcare insurance.

^c^ Migrant status, regardless of legal residency, as recorded by the Chilean health system.

^d^ The natural logarithm of the Weibull shape parameter. ln = natural logarithm. A. Early follow-up was defined as the first six months after SARS-CoV-2 infection identification, capturing incident TB cases occurring within this period. B. Extended follow-up was defined as up to three years after SARS-CoV-2 infection identification, capturing incident TB cases occurring within this period. All statistical tests were two-sided.

To address potential bias from early TB detection, we conducted sensitivity analyses excluding TB events occurring within 30, 60, 90, and 180 days after SARS-CoV-2 infection. A progressive attenuation of effect estimates was observed with increasing exclusion thresholds, while associations for covariates remained stable (Table S3).

In stratified analysis by pre- and post-RECOVERY trial periods, the association strengthened among unvaccinated individuals following the introduction of corticosteroid-based treatment for severe COVID-19 pneumonia. Among unvaccinated individuals requiring hospitalization, the aHR for incident TB increased from 8.78 (95% CI: 7.16–10.75) in the pre-RECOVERY period to 15.64 (95% CI: 11.89–20.56) in the post-RECOVERY period. Similarly, among ICU-admitted patients, the aHR rose from 8.13 (95% CI: 6.59–10.05) to 16.06 (95% CI: 12.18–21.17). In contrast, fully vaccinated individuals exhibited substantially lower risks of new-onset TB even in the post-RECOVERY period (aHR of 2.36 [95% CI: 1.58–3.52] for hospitalized patients; aHR 2.80 [95% CI: 1.76–4.45] for ICU-admitted patients) (Table 4).

**Table 4:** Factors associated with new-onset TB at one year of follow-up stratified by period relative to RECOVERY trial publication date (pre- and post-implementation of corticosteroids and immunomodulators), and SARS-CoV-2 vaccination status (Chile, 2020-2022)

	A. Pre-RECOVERY Trial ^a^		B. Post-Recovery Trial ^a^ (COVID-19 unvaccinated) ^b^		C. Post-Recovery Trial ^a^ (COVID-19 vaccinated) ^b^	
	Crude HR (CI 95%)	Adjusted HR (CI 95%)	Crude HR (CI 95%)	Adjusted HR (CI 95%)	Crude HR (CI 95%)	Adjusted HR (CI 95%)
TB cases (N/ person-years)	95/ 245,190.90	–	389/ 2,788,880.5	–	249/ 571,446.31	–
TB incidence rate per 100,000 person-years	38.75 (31.35-47.36)	–	13.95 (12.6–15.4)	–	43.57 (38.33–49.33)	–
COVID-19 severity						
Non-hospitalized	Ref	Ref	Ref	Ref	Ref	Ref
Hospitalized	10.58 (8.73–12.83)	8.78 (7.16–10.75)	17.46 (13.45–22.65)	15.64 (11.89–20.56)	3.34 (2.28–4.92)	2.36 (1.58–3.52)
ICU-admitted	11.12 (9.27–13.33)	8.13 (6.59–10.05)	18.80 (14.90–23.73)	16.06 (12.18–21.17)	4.76 (3.12–7.28)	2.80 (1.76–4.45)
Diabetes	2.86 (2.32–3.53)	1.24 (0.99–1.56)	3.02 (2.40–4.25)	1.21 (0.89–1.65)	1.90 (1.26–2.85)	1.14 (0.74–1.75)
Major chronic comorbidities ^c^	4.44 (3.57–5.52)	2.30 (1.82–2-89)	5.13 (3.83–6.87)	2.37 (1.74–3.23)	3.07 (2.05–4.57)	2.13 (1.40–3.24)
Age (years)	1.02 (1.01–1.02)	1.06 (0.99–1.13)	1.02 (1.01–1.02)	0.99 (0.99–1.00)	1.01 (1.01–1.02)	1.00 (0.99–1.00)
Sex (Male)	1.81 (1.56–2.10)	1.74 (1.49–2.02)	2.05 (1.66–2.53)	2.01 (1.62–2.48)	2.48 (1.93–3.18)	2.46 (1.90–3.19)
Public health insurance^d^	3.47 (2.55–4.73)	2.90 (2.13–3.96)	6.52 (3.52–11.88)	5.38 (2.95–9.83)	3.42 (2.23–5.26)	3.37 (2.19–5.21)
Migrant ^e^	3.00 (2.48–3.63)	3.43 (2.82–4.17)	3.09 (2.39–3.99)	3.46 (2.65–4.51)	4.18 (2.78–6.28)	4.85 (3.22–7.30)
ln (p) ^f^	–	−0.52 (−0.72– −0.32)	–	−0.56 (−0.66– −0.46)	–	−0.18.(−0.31– −0.06)

*TB* Tuberculosis. *CI*: Confidence Interval. N persons years: events (New-onset TB /time of follow-up in years. Rate per 100,000 person-years: events (new-onset TB) divided by follow-up time in years multiplied by 100,000 and its confidence interval (95%), *Ref*. Reference category, *HR* Hazard ratios, *ICU* Intensive care units.

^a^ The RECOVERY trial (published June 2020) marked the beginning of widespread corticosteroid use in COVID-19 management. Given the known association between corticosteroids and TB reactivation, analyses were stratified into pre- and post-publication periods. The RECOVERY trial (published June 2020) marked the beginning of widespread corticosteroid use in COVID-19 management. Analyses were stratified into three periods: A) pre-RECOVERY period, defined from the start of the pandemic (1 January 2020) to 30 June 2020; B) post-RECOVERY period among unvaccinated individuals, defined from 1 July 2020 onwards; and C) post-RECOVERY period among vaccinated individuals, defined from 1 July 2020 onwards among those who had completed a SARS-CoV-2 vaccination scheme before infection.

^b^ SARS-CoV-2 vaccination status was considered complete if individuals had received two doses of any SARS-CoV-2 vaccine, regardless of vaccine type or combination (including Pfizer-BioNTech [BNT162b2], Sinovac [CoronaVac], AstraZeneca [ChAdOx1-S], or mixed schedules), or a single dose of the CanSino (Ad5-nCoV) vaccine, prior to the SARS-CoV-2 infection identification.

^c^ At least one major chronic comorbidity (Lung, kidney or liver disease) was reported at the time of SARS-CoV-2 identification.

^d^ Belong to the Chilean public healthcare insurance system.

^e^ Migrant status, regardless of legal residency, as recorded by the Chilean health system^.^ The natural logarithm of the Weibull shape parameter. ln = natural logarithm. All statistical tests were two-sided.

Finally, in an age-stratified analyses, the association between severe COVID-19 and incident TB was stronger among individuals younger than 50 years compared to those aged 50 years or older. For COVID-19 requiring hospitalization the aHR was 12.75 (95% CI 9.69–16.77) in those <50 years versus 6.17 (95% CI 4.63–8.23) in those ≥50 years. Among ICU-admitted patients, the corresponding aHR were 9.53 (95% CI 6.49–14.01) and 7.15 (95% CI 5.53–9.24), respectively (Table S4).

Across multiple sensitivity analyses—including the fully adjusted Weibull, Cox proportional hazards, and Fine and Gray competing risks models—effect estimates were consistent, supporting the robustness of our findings to model specification (Tables S5–S7).

## Discussion

We found that severe COVID-19, in this nationwide cohort study, was associated with an increased risk of new-onset TB within one year compared with non-severe disease. Although the risk declined over time, particularly after six months, it remained elevated, suggesting that both the acute and post-acute phases of COVID-19 may impair host immunity and lung integrity, thereby facilitating TB development.

Several immunopathological mechanisms could explain this association. Acute COVID-19 triggers intense pulmonary inflammation and tissue damage, potentially creating a permissive environment for reactivation of latent *M. tuberculosis* infection^8,18^. SARS-CoV-2 may disrupt granuloma integrity, leading to mycobacterial reactivation^7^. In vitro studies have detected high levels of pro-inflammatory cytokines and chemokines in tuberculoma walls up to three months post-COVID-19, suggesting a persistent inflammatory response^19^. Moreover, type I interferons (IFN), which are essential for antiviral immunity, may paradoxically impair *M. tuberculosis* control^20^. Viral co-infections and chemical IFN inducers exacerbate *M. tuberculosis* disease in murine models^21^. This phenomenon may be mediated by the suppression of *M. tuberculosis*-specific IFN-γ responses, resulting in increased bacterial burden and exacerbated pulmonary inflammation.

Although crude analyses initially suggested a higher incidence of TB among SARS-CoV-2 - vaccinated individuals, this finding was likely explained by confounding, as age and comorbidities were the main determinants of vaccination prioritization in Chile. Vaccination significantly modified the association between severe COVID-19 and subsequent tuberculosis risk, attenuating the risk among vaccinated individuals compared with their unvaccinated counterparts. Importantly, these findings should not be interpreted as evidence that SARS-CoV-2 vaccination directly prevents TB. Rather, SARS-CoV-2 vaccination acts upstream by reducing the likelihood of severe COVID-19, the clinical factor most strongly associated with subsequent TB risk. Support for this hypothesis comes from a nationwide cohort study in Taiwan showing lower TB incidence among older adults who received the influenza vaccination compared to those unvaccinated (145.2 vs. 175.5 cases per 100,000 person-years; *p* = 0.002)^22^. Beyond reducing the severity of specific viral infections, vaccines may also confer broader protection against unrelated pathogens through heterologous or trained immunity mechanisms^23^.

Over one-third of TB cases of the cohort were diagnosed within two months of SARS-CoV-2 infection, and time-restricted analyses showed that excess risk was greatest within the first 6 months after severe COVID-19. This early clustering may reflect multiple processes: reactivation of TBI triggered by immune disruption, accelerated progression from subclinical to clinical TB, or increased clinical surveillance during post-COVID evaluations. Subclinical TB can persist undetected for prolonged periods^24,25^, and pandemic-related disruptions in TB services may have delayed diagnoses^26^, contributing to the early concentration of cases. Excluding early events (30–180 days) attenuated estimates, but associations persisted, suggesting that findings are not solely due to early detection. The attenuation of the association over longer follow-up is consistent with recovery of immune function and the influence of survivor bias and competing risks.

Using publication of the RECOVERY trial as a proxy for widespread adoption of corticosteroids and immunomodulators in COVID-19 pneumonia care, we observed that TB risk nearly doubled in the post-RECOVERY period^27^. This increased risk likely reflects corticosteroid-induced immunosuppression, a known risk factor for TBI reactivation^16^. Additionally, in the post-RECOVERY period, survival gains from corticosteroid use may have extended follow-up and revealed a higher incidence of new-onset TB after critical COVID-19^27^. Interestingly, ICU-admitted individuals had slightly lower hazard ratios than hospitalized non-ICU patients, likely reflecting survivor bias, as early mortality may have truncated follow-up in the most critically ill patients.

Beyond COVID-19 severity, we identified established TB risk factors, including male sex, migrant status, enrollment in the public healthcare system, and chronic comorbidities such as diabetes, liver, lung, and kidney disease^5,24,28^. Prior work along the Mexico–U.S. border reported nearly eightfold higher TB odds among individuals with both diabetes and prior COVID-19^29^. Emerging evidence suggests a possible synergistic interaction between COVID-19, diabetes, and TB, particularly given reports of new-onset diabetes following SARS-CoV-2 infection. This triad may represent an emerging challenge for TB control in high-burden settings^30^.

The mechanisms linking severe COVID-19 and TB are not yet fully understood. Our findings support two non-mutually exclusive hypotheses. First, severe SARS-CoV-2 infection may promote *M. tuberculosis* reactivation through immune dysregulation (e.g., lymphopenia, impaired interferon signaling), corticosteroid-induced immunosuppression, and structural lung damage. Second, severe COVID-19 may function as a marker of underlying biological or social vulnerability predisposing individuals to TB. Both mechanisms may operate concurrently. Given that recent estimates suggest that nearly one in four (24.8%, 95% CI: 19.7–30.0%) of the global population has TBI, even modest increases in reactivation risk could have substantial public health implications^17^.

While causal inference is limited by the observational nature of our study, several of Bradford Hill’s viewpoints are met^31^. The strength and consistency of the associations across models and sensitivity analyses support the robustness of our findings. Biological plausibility aligns with known immunopathological mechanisms. While a dose-response gradient was not evident, this may have been obscured by survivor bias among ICU patients. Other criteria, including specificity and experimental evidence, remain to be established. Nonetheless, our results provide a compelling rationale for further mechanistic and longitudinal studies to test these hypotheses.

This study has some limitations. First, residual confounding cannot be excluded. Behavioral TB risk factors, such as smoking, alcohol misuse, and housing conditions^24^, were unavailable, and although public healthcare insurance status served as a proxy for socioeconomic vulnerability^32^, it is an imperfect substitute. Second, individual-level data on corticosteroid and immunomodulator exposure were unavailable. Calendar period was therefore used as a proxy for changes in the standard of COVID-19 pneumonia care, which may introduce exposure misclassification and is subjected from ecological fallacy. Consistent findings across pre- and post-RECOVERY periods support the robustness of the main results but should not be interpreted as evidence of individual-level treatment effects. Third, while the study aimed to capture the new-onset TB following SARS-CoV-2 infection, some individuals may have had subclinical TB at baseline. Yet, consistent associations in sensitivity analyses after excluding early follow-up periods reinforce the temporal relationship. Fourth, migrant populations may be underrepresented due to systemic barriers to healthcare access, which could lead to an underestimation of TB risk in this group^33^. Fifth, analyses were restricted to the first documented SARS-CoV-2 infection per individual, and did not account for specific SARS-CoV-2 variants or pandemic waves, which may limit generalizability; however, the large sample size likely mitigates this issue. Sixth, comorbidities were assessed only at the time of SARS-CoV-2 diagnosis and not longitudinally, potentially missing evolving risk profiles. Importantly, individuals with known immunocompromising conditions, including HIV infection and those receiving immunosuppressive therapies at the time of SARS-CoV-2 identification, were excluded from the study to avoid overestimating TB risk, as immunosuppression is a well-established driver of TBI reactivation. Seventh, differential detection bias is possible, as hospitalized individuals undergo more intensive clinical assessments. In contrast, non-hospitalized patients may have experienced diagnostic delays. To address this, we extended follow-up to more than three years, allowing adequate time for new-onset TB across all COVID-19 severity groups. Although this strategy cannot fully correct for differences in diagnostic intensity, it reduces systematic variation in detection opportunity. Finally, a small proportion of participants (1.51%) were lost to follow-up, primarily due to non-TB deaths. To account for the competing risk of non-TB mortality, we fitted a Fine and Gray model (Table S8), yielding estimates like those from our Weibull analysis. Given the known link between COVID-19 severity and early mortality, some TB cases may have gone undiagnosed, potentially underestimating the true association. Additionally, as with any study using administrative data, potential misclassification or missing data in national records could have introduced bias, though such limitations would likely lead to conservative risk estimates rather than overestimation. Vaccination status was modeled as a binary variable, and vaccine-specific effects were not examined due to heterogeneity in vaccine platforms, dosing schedules, and administration timing across the study population. Nonetheless, we observed clear effect modification by vaccination status, which suggests that vaccination may partially mitigate the downstream consequences of SARS-CoV-2 infection relevant to TB pathogenesis by modulating immune responses or attenuating infection severity, warranting further immunological research.

In summary, this nationwide study demonstrates a markedly increased risk of new-onset TB following severe COVID-19, particularly among COVID-19-unvaccinated individuals and after widespread adoption of corticosteroid use. Risk is highest in the first year after infection but remains elevated thereafter. These findings support integrating TB prevention and COVID-19 care strategies, especially in high TB-burden settings. Targeted screening for TBI and active TB disease among survivors of severe COVID-19, particularly those who are unvaccinated or have comorbidities such as diabetes mellitus, may help mitigate downstream TB burden. Importantly, our findings also have broader implications for future viral pandemics. Severe respiratory viral infections, particularly those associated with intense inflammation, immune dysregulation, or immunomodulatory treatment, may transiently increase the risk of reactivation of latent infections such as TBI. Integrating surveillance systems, ensuring continuity of TB services, and incorporating targeted screening into post-viral care pathways may reduce indirect mortality in future global health crises.

## Methods

### Study design and data

We conducted a nationwide retrospective cohort study in Chile (population ~19.5 million), spanning the period from March 1, 2020, to October 28, 2022. We used routinely collected clinical and epidemiological data from national surveillance systems. Five national-level registries maintained by the Chilean Ministry of Health (Ministerio de Salud, MINSAL) were integrated for this analysis, covering both public and private healthcare sectors across all levels of care and providing near-complete population coverage: i) The National Registry of SARS-CoV-2 Infections; ii) National TB Program Notification System; iii) The National Registry of Hospital Discharges; iv) The National Mortality Registry and v) The National SARS-CoV-2 vaccination Registry.

Records were deterministically linked using the Chilean national identification number (‘*Rol Único Nacional*’), enabling longitudinal follow-up across data sources. Additional details regarding the structure, variables, and data management procedures for these registries are provided in the Supplementary Material.

### Study population

We built a nationwide cohort of adults aged ≥18 years with a first laboratory-confirmed SARS-CoV-2 infection. Subsequent SARS-CoV-2 infections were not considered because of the cumulative immunological effect, which could overestimate the association size^34^. The majority (*n* = 2,776,491; 76.97%) were confirmed by reverse transcription polymerase chain reaction (RT-PCR), and the remaining 23.02% through antigen testing.

The date of SARS-CoV-2 infection served as the index date for follow-up. We excluded individuals who (i) died on the same day as SARS-CoV-2 infection diagnosis, (ii) had a record of TB notification prior to their SARS-CoV-2 diagnosis, (iii) were aged ≥100 years (to reduce potential data entry errors), or (iv) had documented immunocompromising conditions (e.g., HIV infection, cancer) or were receiving immunosuppressive therapy, at the time of SARS-CoV-2 diagnosis. Immunosuppression status (yes/no) did not include treatments initiated subsequently for acute COVID-19 management.

**Fig. 2:**
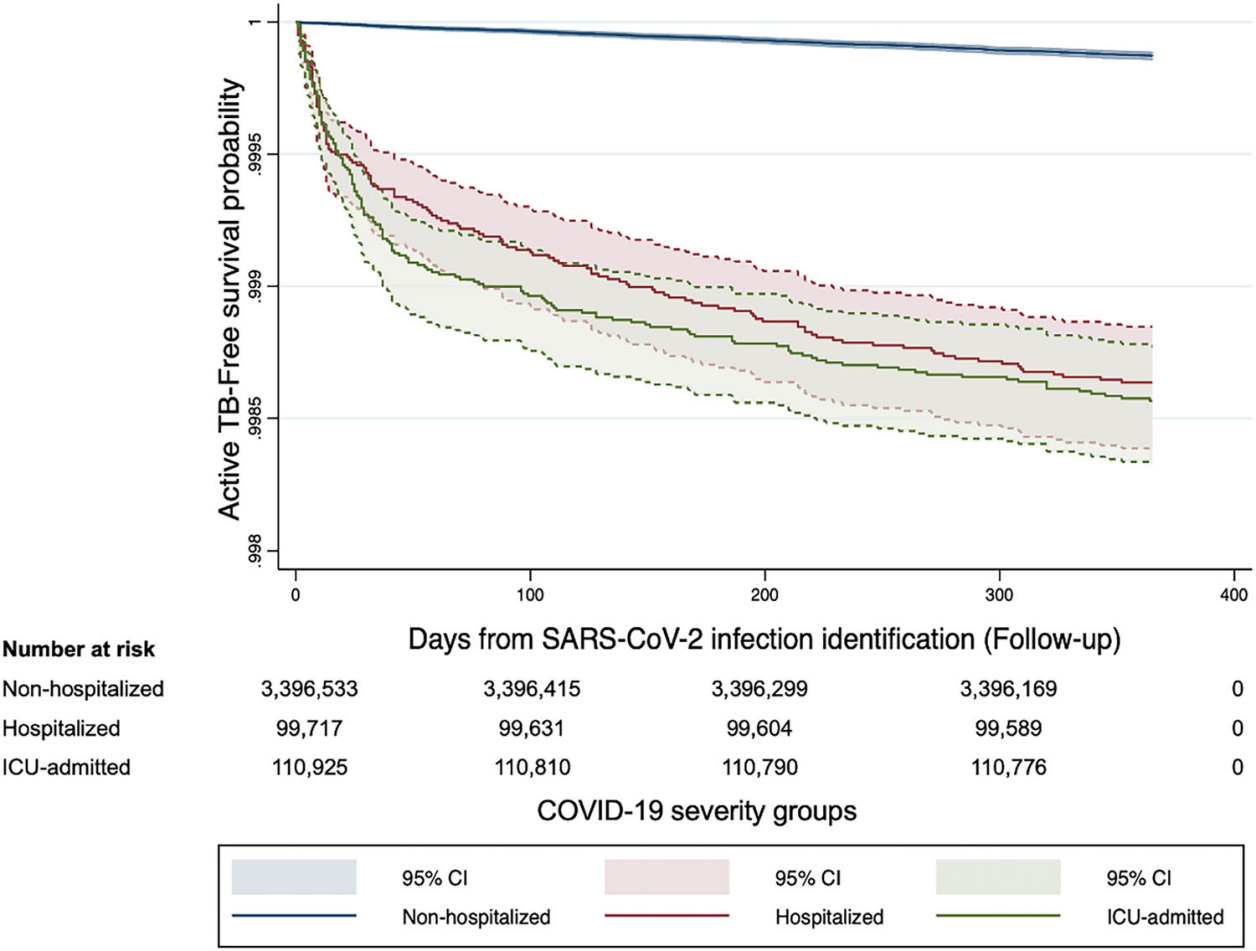
Kaplan-Meier time-to-new onset TB curves across COVID-19 severity groups. Kaplan-Meier time-to-event curves for risk of new-onset TB across COVID-19 severity groups. Notes: Differences between groups were assessed using the log-rank test (two-sided). No adjustment for multiple comparisons was performed. CI confidence intervals. ICU intensive care unit, TB tuberculosis.

A detailed flowchart of the inclusion and exclusion criteria is provided in Fig. 2.

### Exposure variable

COVID-19 severity was defined hierarchically based on the highest level of care reached during the index infection, treated as a fixed exposure, and categorized into three mutually exclusive exposure groups: First, non-hospitalized: individuals who tested positive for SARS-CoV-2 (symptomatic or asymptomatic) and did not required hospitalization for COVID-19-related causes. Second, hospitalized: individuals who tested positive for SARS-CoV-2 and had a recorded hospitalization and discharge for COVID-19-related causes, without intensive care unit (ICU) admission. Third, ICU-admitted: individuals who tested positive for SARS-CoV-2 and were admitted to an ICU for COVID-19-related complications.

### Study outcome

The outcome was new-onset TB (TB diagnosis in Chile is based on Xpert MTB/RIF Ultra since 2019)^35^, defined as any newly recorded case (pulmonary or extrapulmonary) in the National Tuberculosis Program Registry, during the follow-up period after a SARS-CoV-2 diagnosis.

Hospital discharge and mortality national records were also used to capture cases with TB listed as a discharge diagnosis or cause of death. New-onset TB was defined from the date of SARS-CoV-2 diagnosis up to one year of follow-up.

### Independent variables

Sociodemographic and clinical independent variables were obtained from the national SARS-CoV-2 infections and SARS-CoV-2 vaccination registries and included three subgroups: First, pre-existing conditions were classified into three categories based on information recorded at the time of SARS-CoV-2 infection identification. Diabetes mellitus was analyzed as a separate category. Major chronic comorbidities included kidney, liver, or chronic pulmonary diseases. Other comorbidities were defined as the presence of at least one of the following conditions: cardiovascular disease, neurological disorders, obesity, hypertension, or other unspecified chronic conditions. Second, socio-demographic variables: age, sex (as recorded in national health registries), healthcare insurance type (public vs. private), migrant status (regardless of legal status), indigenous status, and healthcare worker status. Third, SARS-CoV-2 vaccination status which was classified as complete if, prior to the infection, an individual had received a full primary vaccination scheme, defined as two doses of any SARS-CoV-2 vaccine, regardless of vaccine type or combination (including Pfizer-BioNTech [BNT162b2], Sinovac [CoronaVac], AstraZeneca [ChAdOx1-S], or mixed schedules), or a single dose of the CanSino (Ad5-nCoV) vaccine, in accordance with the national vaccination guidelines in Chile.

### Statistical analysis

We conducted a descriptive analysis of the national SARS-CoV-2 infection cohort, stratified by the outcome variable (new-onset TB vs. non-TB). We also stratified by SARS-CoV-2 vaccination status (vaccinated vs. unvaccinated). Categorical variables were presented as frequencies and percentages; continuous variables were summarized using medians with interquartile ranges (IQR). We used Pearson’s Chi-squared test, Wilcoxon rank-sum test, or Fisher’s exact test for group comparisons, as appropriate. All statistical tests were two-sided. Exact *p*-values are reported unless *p* < 0.0001.

Person-time at risk was calculated in days from the date of SARS-CoV-2 diagnosis (index date) to the earliest of new-onset TB, death, or end of follow-up. TB incidence rates were expressed per 100,000 person-years, with 95% confidence intervals (CIs). Furthermore, we generated Kaplan–Meier time-to-TB curves across categories of COVID-19 severity; group comparisons were conducted using the log-rank test to evaluate curve differences. Time-to-event analyses were performed using parametric Weibull regression to estimate cause-specific (conditional) crude and adjusted hazard ratios for incident TB. These models quantify the instantaneous risk of TB among individuals who remained alive and TB-free over follow-up, with death due to COVID-19 or other causes treated as a competing event and handled through censoring. Weibull models were selected to accommodate non-proportional hazards, as the proportional hazards assumption was violated in Cox regression, and the shape parameter (ln[*p*]) allowed the hazard function to vary flexibly over time. Independent variables included in the models were selected based on biological plausibility and clinical and epidemiological considerations. To guide model specification and explore potential confounding structures, we constructed a directed acyclic graph (DAG), included in the Supplementary Materials (Fig. S2). We first examined two-way potential interactions among independent variables and subsequently assessed effect modification by SARS-CoV-2 vaccination in the association between COVID-19 severity and new-onset TB (Table S9). Because SARS-CoV-2 vaccination is an antecedent of COVID-19 severity and not known to be independently associated with TB, we stratified Weibull models by vaccination status. We also performed an age-stratified analysis ( < 50 years and ≥50 years), based on clinical relevance, comparing adjusted effects to minimize residual confounding.

### Sensitivity and robustness analyses

We conducted multiple additional analyses to assess the robustness of our findings. First, we extended follow-up beyond one year, incorporating all available person-time (up to more than three years) to assess long-term associations between COVID-19 severity and new-onset TB. Second, we excluded individuals with ≤30, ≤60, ≤90, or ≤180 days of follow-up to reduce potential bias from undiagnosed pre-existing active TB at the time of SARS-CoV-2 infection (Table S3)

To explore the potential influence of corticosteroid and immunomodulator use for severe COVID-19 pneumonia, we stratified the cohort by calendar period: pre-RECOVERY trial (March-June 2020) and post-RECOVERY trial (July 2020 onward), based on the trial’s first publication date (June 22, 2020)^27^. Within the post-RECOVERY period, we performed two additional analyses: (i) excluding individuals with a complete SARS-CoV-2 vaccination scheme—to align with the pre-RECOVERY population and reduce confounding from vaccine-induced protection—and (ii) including only fully vaccinated individuals to explore the interaction between possible corticosteroid exposure and SARS-CoV-2 vaccination status on TB risk.

To evaluate the robustness of the findings in the presence of competing risks (deaths due to COVID-19 or other causes), Fine and Gray subdistribution hazard models were additionally fitted as a sensitivity analysis. (Table S8).

Regressions were conducted using complete-case analysis, as implemented by default in Stata. Given the extremely low proportion of missing data, no multiple imputation was performed. All statistical analyses were conducted using Stata version 17 (StataCorp LLC, College Station, TX, USA).

### Ethical considerations

Individual-level data were anonymized by the Department of Epidemiology, MINSAL, prior to analysis, ensuring that no personally identifiable information was accessible. The study was approved by the Ethics Research Committee of the Pontificia Universidad Católica de Chile (ID 220418009). The funder had no role in study design, data collection, analysis, interpretation, or report writing.

### Reporting summary

Further information on research design is available in the Nature Portfolio Reporting Summary linked to this article.

## Supplementary information

Supplementary analyses and dataset information

Reporting Summary

Transparent Peer Review file

## Source data

Data Source

## Data Availability

The individual-level data used in this study are derived from national health registries in Chile, including the National Tuberculosis Registry, the National SARS-CoV-2 Infection Registry, the National Hospital Discharge Registry, and the National Death Registry. These data contain sensitive personal health information and are protected under Chilean Law No. 19,628 on the Protection of Private Life and regulations from the Chilean Ministry of Health (MINSAL). For this reason, the raw individual-level data are not publicly available and cannot be deposited in a public repository. Access to these data is restricted to authorized researchers and requires approval from MINSAL and relevant institutional ethics committees. Researchers seeking access must submit a formal data request to the Department of Epidemiology at MINSAL, including a study protocol and proof of ethical approval. Access is granted only for non-commercial research purposes and under data use agreements that prohibit data sharing or redistribution. Aggregated and anonymized source data underlying the main figures and tables are provided with this paper. Source data are provided with this paper.

## Code availability

The analytical code used to generate the results and figures in this study is available from the corresponding author upon request. All analyses were conducted using Stata (version 17; StataCorp LLC, College Station, TX, USA). No novelty algorithms were developed.
