# Supplementary material for: Risk of incident tuberculosis after severe COVID-19: a nationwide cohort study
Source: Nat Commun. 2026 Jun 29;17:8124. doi: 10.1038/s41467-026-74528-5 (PMC13458518; doi:10.1038/s41467-026-74528-5)
Supplement: Supplementary file 1 [file 41467_2026_74528_MOESM1_ESM.pdf]

Supplementary Information

Risk of incident tuberculosis after severe COVID-19: a nationwide cohort study

Salvador Vargas-García Ph.D. (c)[1,2,3], Lourdes García-García M.D, DrSc[4], Christian García Ph.D.[5,6], Natalia Vergara MS[7], Nadia Escobar MD, MSc[8], Luis A. Villarroel Ph.D.[2], Kasim Allel Ph.D.[3,9,10], Eduardo A. Undurraga Ph.D.[3,11*+], María Elvira Balcells MD, MSc[3,10*+]

1. *School of Public Health, Pontificia Universidad Católica de Chile, Chile*
2. *Universidad Autónoma de Ciudad Juárez, Instituto de Ciencias Biomédicas, México*
3. *SENTINET: Surveillance, Epidemiology, and New Technologies for Infectious Emerging Threats, Chile*
4. *Centro de Investigación sobre Enfermedades Infecciosas (CISEI), Instituto Nacional de Salud Pública, México*
5. *Programa Centro de Salud Pública, Universidad de Santiago, Chile*
6. *Servicio de Salud Metropolitano Oriente, Chile.*
7. *Department of Epidemiology, Ministry of Health, Chile*
8. *National Tuberculosis Control and Elimination Program, Ministry of Health, Chile*
9. *Nuffield Department of Primary Care Health Sciences, University of Oxford, United Kingdom*
10. *Department of Infectious Diseases, School of Medicine, Pontificia Universidad Católica de Chile, Chile*
11. *Escuela de Gobierno, Pontificia Universidad Católica de Chile, Chile*

+ These authors jointly supervised this work

* Corresponding authors:

María Elvira Balcells (ebalcells@uc.cl)

Eduardo A. Undurraga (eundurra@uc.cl)

Contents

Supplementary Methods 3

Dataset description 3

The Chilean healthcare system 5

Additional descriptive statistics & results (Tables S1-S9) 6

References for the Supplementary Material 16

Supplementary Methods

Dataset description

The study draws on five population databases from the Chilean Ministry of Health (MINSAL), including private and public health and all levels of care. The Unique National Role (RUN), a compulsory identification number uniquely assigned to each person living in Chile, was used to link the databases. This number is primarily used for administrative and tax purposes, as well as personal identification in various procedures, including medical care. Newly arrived migrants in Chile can request a provisional RUN to access medical care and be recorded in the aforementioned information systems. We used routinely collected clinical and epidemiological data from national surveillance systems. The primary data source was EPIVIGILA, Chile's national notifiable disease reporting system. This platform ensures timely, centralized, high-quality data collection for disease surveillance, control, clinical monitoring, case traceability, early warning, and outbreak detection. [1]

(i) National TB Registries: TB is a mandatory daily notification disease in Chile, reported primarily by the attending physician to the Epidemiology Department of the Chilean Ministry of Health (MINSAL) through an epidemiological surveillance electronic platform implemented in 2019 (EPIVIGILA). All clinical and epidemiological information for confirmed cases is later validated and consolidated within the National Control and Elimination of Tuberculosis Program (PROCET), which maintains a registry of all TB case reports, covering both new cases and relapses (pulmonary and extrapulmonary), sociodemographic characteristics, treatment status, drug resistance, main comorbidities, and risk factors. The period analyzed in the present study covers January 1, 2020, to December 31, 2023.

(ii) National Registry of SARS-CoV-2 Infection: Starting from March 1, 2020, to November 30, 2022, the records of confirmed SARS-CoV-2 infections were also captured on the EPIVIGILA platform and centralized in the Epidemiology Unit of MINSAL. These notifications were compulsory for the attending physicians and covered epidemiological information for the index case, as well as its contacts, the clinical course of SARS-CoV-2 infection, both asymptomatic and symptomatic cases registered (symptoms, requirement for supplemental oxygen, record of hospitalization and admission to the intensive care unit with or without the need for assisted mechanical ventilation, main patient comorbidities and death during hospitalization). The information included in this study covers the period from March 3, 2020, to October 28, 2022.

(iii) National Registry of Hospital Discharges: This database is centralized in the Department of Statistics and Health Information (DEIS) from the MINSAL and contains the morbidity and mortality reports for all hospital discharges at the national level. For this study, all hospital discharges were considered from May 29, 2019, to December 31, 2023; the primary and, where appropriate, secondary discharge diagnoses were considered using the International Classification of Diseases, Tenth Revision (ICD-10). Those responsible for directly capturing the clinical history are healthcare workers, primarily physicians.

(iv) National Death Registry: The information regarding the cause of death was obtained from DEIS between January 1, 2020, and December 31, 2023, and the International Classification of Diseases, Tenth Revision (ICD-10) was used to record the diagnosis of death (both primary and secondary) on the death certificate, made by a physician. The Civil Registry of Chile subsequently validates this information. Mortality data in Chile have historically shown high completeness and validity.

(v) National Registry of SARS-CoV-2 Vaccination: This database compiled information on all vaccine doses administered to the general population in Chile, including the date, doses, and type of vaccine from December 25, 2020, to October 28, 2022. This information is captured and validated by the DEIS of MINSAL. SARS-CoV-2 Vaccination had national coverage, and Chile reached some of the highest rates globally, including boosters (homologous and heterologous).[2] By June 23, 2021, Chile reported that more than 80% of the population was vaccinated.[3]

The Chilean healthcare system

Chile has some of the strongest health indicators in Latin America and within its income category.[4,5] A recent global comparison using an index of effective universal healthcare coverage, weighted by potential health gains, placed Chile in the 74th percentile, positioned between South American countries such as Argentina, Brazil, and Uruguay, and high-income countries such as Israel and the United States.[6]

The Chilean health system provides near-universal coverage (almost 98%).[5] It is a dual and segmented system comprising a public solidarity-based regime administered by the Fondo Nacional de Salud (FONASA) and an individual, insurance-based private regime managed by the Instituciones de Salud Previsional (ISAPRE). In 2014, FONASA insured 75·2% of the population[7], primarily low-income individuals with higher age and prevalence of chronic diseases, while ISAPREs served 18·5%, mainly high-income young men with lower health risks, thus segmenting the system by risk and income.[8]

All formal sector workers contribute 7% of taxable income to health insurance, with contributions subject to an upper cap (roughly equivalent to ~USD 2,000 per month in recent years). They can allocate this 7% to FONASA or any of the more than 20 open ISAPREs; eligibility and plan choice in ISAPREs depend on whether the mandatory 7% covers the plan price (with risk-rated premiums historically used in ISAPREs). A worker's ability to join an ISAPRE depends on how much their 7% can cover. The private sector includes for-profit and nonprofit clinics, diagnostic centers, and private offices. Public provision is through municipal health centers and the National Health Services System (SNSS) public hospitals, offering primary, secondary, and tertiary care.[3] FONASA does not discriminate based on age, gender, income, number of dependents, pre-existing conditions, or nationality. Access to medical care is available through a network of primary care centers and referral hospitals.[8]

In practice, ISAPREs primarily insure middle- to high-income individuals, whereas FONASA covers low- to middle-income groups. FONASA, or the chosen ISAPRE, provides health insurance coverage to workers and their legal dependents in exchange for the mandatory contribution.[4]

Additional descriptive statistics & results (Tables S1-S9)

Table S1. Characteristics of the SARS-CoV-2 infection national cohort according to SARS-CoV-2 Vaccine status

	All individuals	COVID-19 Unvaccinated [a]	COVID-19 Vaccinated [a]	p-value [b]
	N=3,607,175	3,013,241 (83.53%)	593,934 (16.46%)	
**COVID-19 severity (%)**				<0.0001
Non-hospitalized	3,396,533 (94.16)	2,863,262 (95.04)	593,271 (89.78)	
Hospitalized	99,717 (2.76)	67,768 (2.24)	31,949 (5.37)	
ICU-admitted	110,925 (3.07)	82,211 (2.72)	28,714 (4.83)	
**Diabetes (%)**	188,467 (5.22)	147,316 (4.88)	41,151 (6.92)	<0.0001
**Major chronic comorbidities [c] (%)**	114,216 (3.16)	89,295 (2.96)	24,921 (4.19)	<0.0001
**No illness reported (%)**	1,661,032 (46.04)	1,417,763 (47.05)	243,269 (40.95)	<0.0001
**Age, median (IQR)**	40 (29-55)	40 (29-54)	42 (32-59)	<0.0001
**Sex, Male (%)**	1,689,895 (46.84)	1,532,958 (50.87)	211,040 (35.53)	<0.0001
**Public healthcare insurance [d] (%)**	2,984,697 (82.74)	2,513,050 (83.40)	451,647 (76.04)	<0.0001
**Migrant [e] (%)**	239,435 (6.63)	218,603 (7.25)	20,832 (3.50)	<0.0001
**Indigenous status [f] (%)**	86,402 (2.33)	74,759 (2.48)	11,643 (1.96)	<0.0001
**Healthcare worker (%)**	143,453 (3.97)	116,280 (3.85)	27,273 (4.57)	<0.0001

TB: tuberculosis.
SARS-CoV-2 vaccination status was considered complete if individuals had received two doses of any SARS-CoV-2 vaccine, regardless of vaccine type or combination (including Pfizer-BioNTech [BNT162b2], Sinovac [CoronaVac], AstraZeneca [ChAdOx1-S], or mixed schedules), or a single dose of the CanSino (Ad5-nCoV) vaccine, prior to the SARS-CoV-2 infection identification
All statistical tests were two-sided.
[a] Median (IQR) or frequency (%).
[b] Pearson´s Chi-squared test; Wilcoxon rank sum test; Fisher´s exact test.
[c] At least one major chronic comorbidity (Lung, kidney, or liver disease) registered at the time of SARS-CoV-2 identification.
[d] Belong to the Chilean public healthcare insurance.
[e] Migrant status, regardless of legal residency, as recorded by the Chilean health system.
[f] Self-reporting of belonging to an indigenous status.

Table S2. Characteristics of the SARS-CoV-2 infection national cohort according to COVID-19 severity & SARS-CoV-2 Vaccine status.

	All individuals	Non-Hospitalized		Hospitalized		ICU-admitted	
		COVID-19 Unvaccinated [a]	COVID-19 Vaccinated [a]	COVID-19 Unvaccinated [a]	COVID-19 Vaccinated [a]	COVID-19 Unvaccinated [a]	COVID-19 Vaccinated [a]
	N=3,607,175	2,863,262 (84.30%)	533,271 (15.70%)	67,768 (67.96%)	31,949 (32.04%)	82,211 (74.11%)	28,714 (25.89)
**Diabetes (%)**	188,467 (5.22)	117,120 (4.09)	28,079 (5.27)	11,235 (16.58)	5,891 (18.44)	18,961 (23.06)	7,181 (25.01)
**Major chronic comorbidities [b] (%)**	114,216 (3.16)	72,586 (2.54)	17,481 (3.28)	5,596 (8.26)	3,069 (9.61)	11,113 (13.52)	4,371 (15.22)
**Age, median (IQR)**	40 (29-55)	39 (29-53)	41 (31-56)	54 (38-67)	59 (41-72)	65 (52-78)	70 (58-80)
**Sex, Male (%)**	1,689,895 (46.84)	1,396,803 (48.81)	179,098 (33.59)	34,799 (51.35)	15,672 (49.05)	47,251 (57.48)	16,270 (56.66)
**Public healthcare insurance [c] (%)**	2,984,697 (82.74)	2,381,086 (83.16)	398,922 (74.81)	58,221 (85.91)	27,248 (85.29)	73,743 (89.70)	25,477 (88.73)
**Migrant [d] (%)**	239,435 (6.63)	210,202 (7.33)	18,841 (3.53)	4,985 (7.36)	1,201 (3.76)	11,113 (13.52)	4,371 (15.22)
**Follow-up (days), mean (sd)**		364.98 (1.90)	364.92 (4.15)	364.63 (10.62)	364.62 (10.90)	364.62 (10.87)	364.46 (13.03)

TB: tuberculosis. IQR: Interquartile range. sd: standard deviation.

[a] SARS-CoV-2 vaccination status was considered complete if individuals had received two doses of any SARS-CoV-2 vaccine, regardless of vaccine type or combination (including Pfizer-BioNTech [BNT162b2], Sinovac [CoronaVac], AstraZeneca [ChAdOx1-S], or mixed schedules), or a single dose of the CanSino (Ad5-nCoV) vaccine, prior to the SARS-CoV-2 infection identification

[b] At least one major chronic comorbidity (Lung, kidney or liver disease) registered at the time of SARS-CoV-2 identification.

[c] Belong to the Chilean public healthcare insurance.

[d] Migrant status, regardless of legal residency, as recorded by the Chilean health system.

All statistical tests were two-sided.

Table S3. Factors associated with new-onset TB risk at one year at follow-up: sensitivity models excluding the first 30, 60, 90, and, 180 days after SARS-CoV-2 identification, Chile (2020–2022)

	**A. Excluding the first 30 days post-SARS-CoV-2 identification.**		**B. Excluding the first 60 days post-SARS-CoV-2 identification.**		**C. Excluding the first 90 days post-SARS-CoV-2 identification.**		**D. Excluding the first 180 days post-SARS-CoV-2 identification.**	
	**Crude HR (95% CI)**	**Adjusted HR (95% CI)**	**Crude HR (95% CI)**	**Adjusted HR (95% CI)**	**Crude HR (95% CI)**	**Adjusted HR (95% CI)**	**Crude HR (95% CI)**	**Adjusted HR (95% CI)**
**N/ person-years**	551/3,604,254·16	-	472/3,604,244·71	-	431/3,604,236·31	-	285 /3,604,183·81	-
**Incidence rate per 100,000**	15·29 (14·04-16·02)	-	13·10 (11·94-14·33)	-	11·96 (10·86-13·14)	-	7·91 (7·02-8·88)	-
**COVID-19 severity**								
Non-hospitalized	*Ref*	*Ref*	*Ref*	*Ref*	*Ref*	*Ref*	*Ref*	*Ref*
Hospitalized-	6·83 (5·36–8·76)	5·35 (4·14-6·90)	5·93 (4·53-7·77)	4·59 (3·46-6·10)	5·23 (3·90-7·03)	4·18 (3·07-5·69)	4·15 (2·80-6·14)	3·13 (2·08-4·72)
ICU-admitted	6·06 (4·75–7·73)	4·00 (3·03-5·28)	4·65 (3·49-6·19)	3·00 (2·18-4·14)	4·43 (3·27-5·99)	2·99 (2·13-4·20)	3·59 (2·41-5·36)	2·23 (1·44-3·47)
**Diabetes**	2·64 (2·05–3·39)	1·40 (1·06-1·83)	2·44 (1·84-3·22)	1·38 (1·02-1·87)	2·11 (1·55-2·88)	1·24 (0·89-1·73)	2·29 (1·58-3·31)	1·44 (0·97-2·14)
**Major chronic comorbidities** [a]	4·04 (3·15–5·30)	2·55 (1·94-3·35)	3·38 (2·50-4·57)	2·22 (1·62-3·05)	3·13 (2·26-4·33)	2·15 (1·53-3·02)	3·20 (2·15-4·76)	2·32 (1·53-3·51)
**Age** (years)	1·01 (1·01-1·02)	1·00 (1·00-1·01)	1·01 (1·01-1·02)	1·00 (1·00-1·01)	1·01 (1·01-1·02)	1·00 (1·00-1·01)	1·01 (1·00-1·02)	1·00 (1·00-1·01)
**Sex** (Male)	1·74 (1·47–2·07)	1·75 (1·47-2·08)	1·74 (1·44-2·09)	1·77 (1·47-2-13)	1·79 (1·47-2·17)	1·81 (1·49-2·21)	1·83 (1·44-2·32)	1·88 (1·48-2·39)
**Public health insurance** [b]	3·29 (2·32 – 4·66)	2·88 (2·03-4·08)	3·43 (2·39-5·03)	3·08 (2·10-4·53)	3·24 (2·19-4·78)	2·95 (1·99-4·37)	2·87 (1·82-4·52)	2·64 (1·67-4·17)
**Migrant** [c]	2·93 (2·34 – 3·65)	3·23 (2·58-4·06)	2·70 (2·11-3·45)	2·93 (2·28-3·78)	2·77 (2·15-3·57)	2·96 (2·28-3·84)	2·63 (1·91-3·62)	2·84 (2·05-3·94)
ln (p) [d]		0·14 (0·05-0·22)		0·42 (0·33-0·51)		0·56 (0·42-0·66)		1·12 (1·00-1·24)

TB: Tuberculosis. CI: Confidence Interval. N persons years: events (New-onset TB)/time of follow-up in years. Rate per 100,000 person-years: events (New-onset TB) divided by follow-up time in years multiplied by and its confidence interval (95%). Ref. Reference category. HR= Hazard ratios. ICU= Intensive care units [a] At least one major chronic comorbidity (Lung, kidney, or liver disease) reported at the time of SARS-CoV-2 ation. [b] Belong to the Chilean public healthcare insurance. [c] Migrant status, regardless of legal residency, as recorded by the Chilean healthcare system. [d] The natural logarithm of the Weibull shape parameter. All l tests were two-sided.

Table S4. Factors associated with new-onset TB at one year of follow-up: age-stratified sensitivity analysis (<50 years vs ≥50 years), Chile (2020–2022)

	**Younger adults (<50 years)**		**Older individuals (50+ years)**	
	**Crude HR (95% CI)**	**Adjusted HR (95% CI)**	**Crude HR (95% CI)**	**Adjusted HR (95% CI)**
**N/ person-years**	366/2,405,805.62	-	367/1,198,455·04	-
**Incidence rate per 100,000**	15·21 (13·69-16·85)	-	30·62 (27·57-33·92)	-
**COVID-19 severity**				
Non-hospitalized	*Ref*	*Ref*	*Ref*	*Ref*
Hospitalized-	14·41 (11·01–18·87)	12·75 (9·69-16·77)	7·28 (5·51-9·63)	6·17 (4·63-8·23)
ICU-admitted	12·18 (8·40–17·67)	9·53 (6·49-14·01)	8·98 (7·13-11·29)	7·15 (5·53-9·24)
**Diabetes**	3·30 (2·06–5·31)	1·82 (1·11-2·98)	1·99 (1·59-2·44)	1·15 (0·89-1·48)
**Major chronic comorbidities** [a]	2·32 (1·44–3·73)	1·84 (1·14-2·97)	4·72 (3·66-6·09)	2·62 (2·00-3·42)
**Sex** (Male)	1·67 (1·36–2·06)	1·72 (1·39–2·12)	1·97 (1·55-2·54)	1·77 (1·43–2·20)
**Public health insurance** [b]	3·80 (2·47 – 5·84)	3·43 (2·22–5·29)	2·72 (1·77-4·27)	2·34 (1·50–3·64)
**Migrant** [c]	4·67 (3·73–5·83)	4·00 (3·20–5·01)	1·73 (1·09-2·75)	1·96 (1·23–3·12)
ln (p) [d]		-0·37 (0·61--0·41)		-0·51 (0·61--0·41)

Notes. TB: Tuberculosis. CI: Confidence Interval. N persons years: events (New-onset TB)/time of follow-up in years. Rate per 100,000 person-years: events (New-onset TB) divided by follow-up time in years multiplied by 100,000 and its confidence interval (95%). Ref. Reference category. HR= Hazard ratios. ICU= Intensive care units. [a] At least one major chronic comorbidity (Lung, kidney, or liver disease) reported at the time of SARS-CoV-2 identification. [b] Belong to the Chilean public healthcare insurance. [c] Migrant status, regardless of legal residency, as recorded by the Chilean healthcare system. [d] The natural logarithm of the Weibull shape parameter. All statistical tests were two-sided.

Table S5. Factors associated with new-onset TB at one year of follow-up: robustness analysis using a saturated Weibull model including cardiovascular disease status as a Robustness check.

	**Crude HR (95% CI)**	**Adjusted HR (95% CI)**
**N/ person-years**	733/3,604,261	-
**Incidence rate per 100,000**	20·34 (18·89-21·86)	-
**COVID-19 severity**		
Non-hospitalized	*Ref*	*Ref*
Hospitalized	10·58 (8·73–12·83)	8·93 (7·29-10·94)
ICU-admitted	11·12 (9·27–13·33)	8·32 (6·72-10·29)
**Diabetes**	2·86 (2·32–3·53)	1·26 (1·00-1·58)
**Major chronic comorbidities** [a]	4·44 (3·57–5·52)	2·45 (1·94-3·09)
**Age (years)**	1·02 (1·01-1·02)	1·00 (0·99-1·00)
**Sex (Male)**	1·81 (1·56–2·10)	1·74 (1·49-2·02)
**Public healthcare insurance** [b]	3·47 (2·55 – 4·73)	2·91 (2·13-3·97)
**Migrant** [c]	3·00 (2·48 – 3·63)	3·39 (2·78-4·13)
**Cardiovascular disease**	3·57 (2·10-6·07)	0·94 (0·54-1·61)
ln (p) [d]		-0·44 (-0·51- -0·37)

Notes: TB: Tuberculosis. CI: Confidence Interval. N persons years: events (New-onset TB)/time of follow-up in years. Rate per 100,000 person-years: events (New-onset TB) divided by follow-up time in years multiplied by 100,000 and its confidence interval (95%). Ref. Reference category. HR= Hazard ratios. ICU= Intensive care units. [a] At least one major chronic comorbidity (Lung, kidney or liver disease) reported at the time of SARS-CoV-2 identification. [b] Belong to the Chilean public healthcare insurance. [c] Migrant status, regardless of legal residency, as recorded by the Chilean healthcare system. [d] The natural logarithm of the Weibull shape parameter. All statistical tests were two-sided.

Table S6. Factors associated with new-onset TB at one year of follow-up: robustness analysis using a saturated Weibull model including all available variables regardless of biological plausibility or statistical significance, Chile (2020–2022)

	**Crude HR (95% CI)**	**Adjusted HR (95% CI)**
**N/ person-years**	733/3,604,261	-
**Incidence rate per 100,000**	20·34 (18·89-21·86)	-
**COVID-19 severity**		
Non-hospitalized	*Ref*	*Ref*
Hospitalized	10·58 (8·73–12·83)	9·18 (7·49-11·26)
ICU-admitted	11·12 (9·27–13·33)	8·69 (7·01-10·76)
**Diabetes**	2·86 (2·32–3·53)	1·48 (1·16-1·90)
**Major chronic comorbidities** [a]	4·44 (3·57–5·52)	2·45 (1·94-3·09)
**Age (years)**	1·02 (1·01-1·02)	1·00 (0·99-1·00)
**Sex (Male)**	1·81 (1·56–2·10)	1·72 (1·48-2·00)
**Public healthcare insurance** [b]	3·47 (2·55 – 4·73)	2·93 (2·15-4·00)
**Migrant** [c]	3·00 (2·48 – 3·63)	3·36 (2·76-4·09)
**Neurological disease**	3·86 (1·93-7·80)	1·08 (0·53-2·19)
**Heart disease**	2·90 (1·59-5·26)	0·78 (0·42-1·43)
**Hypertension**	1·83 (1·51-2·22)	0·72 (0·57-0·91)
**Obesity**	1·12 (0·66-1·90)	0·57 (0·33-0·98)
**Health care worker**	0·64 (0·40-1·01)	1·11 (0·70-1·76)
**Indigenous status**	1·32 (0·87-1·94)	1·16 (0·77-1·77)
ln (p) [d]		-0·44 (-0·51- -0·37)

Notes: TB: Tuberculosis. CI: Confidence Interval. N persons years: events (New-onset TB)/time of follow-up in years. Rate per 100,000 person-years: events (New-onset TB) divided by follow-up time in years multiplied by 100,000 and its confidence interval (95%). Ref. Reference category. HR= Hazard ratios. ICU= Intensive care units. [a] At least one major chronic comorbidity (Lung, kidney or liver disease) reported at the time of SARS-CoV-2 identification. [b] Belong to the Chilean public healthcare insurance. [c] Migrant status, regardless of legal residency, as recorded by the Chilean healthcare system. [d] The natural logarithm of the Weibull shape parameter. All statistical tests were two-sided.

Table S7. Factors associated with new-onset TB at one year of follow-up: robustness analysis: using traditional Cox model including the same variables selected in the primary Weibull model, Chile (2020–2022)

	**Crude HR (95% CI)**	**Adjusted HR (95% CI)**
**N/ person-years**	733/3,604,261	-
**Incidence rate per 100,000**	20·34 (18·89-21·86)	-
**COVID-19 severity**		
Non-hospitalized	*Ref*	*Ref*
Hospitalized	10·58 (8·73–12·83)	8·92 (7·28-10·94)
ICU-admitted	11·12 (9·27–13·33)	8·31 (6·71-10·28)
**Diabetes**	2·86 (2·32–3·53)	1·26 (1·00-1·58)
**Major chronic comorbidities** [a]	4·44 (3·57–5·52)	2·31 (1·84-2-91)
**Age** (years)	1·02 (1·01-1·02)	1·00 (0·99-1·00)
**Sex** (Male)	1·81 (1·56–2·10)	1·74 (1·49-2·02)
**Public healthcare insurance** [b]	3·47 (2·55 – 4·73)	2·91 (2·13-3·97)
**Migrant** [c]	3·00 (2·48 – 3·63)	3·39 (2·79-4·13)

Notes: TB: Tuberculosis. CI: Confidence Interval. N persons years: events (New-onset TB)/time of follow-up in years. Rate per 100,000 person-years: events (New-onset TB) divided by follow-up time in years multiplied by 100,000 and its confidence interval (95%). Ref. Reference category. HR= Hazard ratios. ICU= Intensive care units. [a] At least one major chronic comorbidity (Lung, kidney, or liver disease) reported at the time of SARS-CoV-2 identification. [b] Belong to the Chilean public healthcare insurance. [c] Migrant status, regardless of legal residency, as recorded by the Chilean healthcare system. All statistical tests were two-sided.

Table S8. Factors associated with new-onset TB at one year of follow-up, Chile (2020-2022). Robustness analysis using a Fine & Gray model considering competing risk.

	**Crude SHR (95% CI)**	**Adjusted SHR (95% CI)**
**N/ person-years**	733/3,604,261	-
**Incidence rate per 100,000**	20·34 (18·89-21·86)	-
**COVID-19 severity**		
Non-hospitalized	*Ref*	*Ref*
Hospitalized	10·58 (8·73–12·83)	8·92 (7·20-11·06)
ICU-admitted	11·12 (9·27–13·33)	8·31 (6·59-10·48)
**Diabetes**	2·86 (2·32–3·53)	1·26 (0·99-1·60)
**Major chronic comorbidities** [a]	4·44 (3·57–5·52)	2·31 (1·82-2·93)
**Age** (years)	1·02 (1·01-1·02)	1·00 (0·99-1·00)
**Sex** (Male)	1·81 (1·56-2·10)	1·74 (1·49-2·02)
**Public healthcare insurance** [b]	3·47 (2·55-4·73)	2·91 (2·13-3·98)
**Migrant** [c]	3·00 (2·48-3·63)	3·39 (2·80-4·11)

Notes.TB: Tuberculosis. CI: Confidence Interval. N persons years: events (New-onset TB)/time of follow-up in years. Ref. Reference category. SHR= Subdistribution Hazard ratios. ICU= Intensive care units. [a] At least one major chronic comorbidity (lung, kidney, or liver disease) reported at the time of SARS-CoV-2 identification. [b] Belong to the Chilean public healthcare insurance. [c] Migrant status, regardless of legal residency, as recorded by the Chilean health system. All statistical tests were two-sided.

Table S9. Factors associated with new-onset TB at one year of follow-up, Chile (2020-2022). Robustness analysis using a Weibull model including interactions between independent variables.

	**Crude HR (95% CI)**	**Adjusted HR (95% CI)**
**N/ person-years**	733/3,604,261	-
**Incidence rate per 100,000**	20·34 (18·89-21·86)	-
**Non-Critical**	8·00 (6·64–9·64)	16·67 (12·99-21·40)
**ICU-admitted**	8·76 (7·35–10·45)	14·64 (11·35-18·88)
**SARS-CoV-2 Vaccine**	3·88 (3·35-4·49)	7·52 (6·21-9·12)
**Non-Critical* SARS-CoV-2 Vaccine**	0·21 (0·14–0·31)	0·13 (0·09-0·20)
**ICU-admitted* SARS-CoV-2 Vaccine**	0·31 (0·21 – 0·45)	0·19 (0·13-0·28)
**Diabetes**	2·86 (2·32–3·53)	1·24 (0·99-1·56)
**Major chronic comorbidities** [a]	4·44 (3·57–5·52)	2·29 (1·82-2·88)
**Age** (years)	1·02 (1·01-1·02)	0·99 (0·99-1·00)
**Sex** (Male)	1·81 (1·56–2·10)	2·08 (1·78-2·42)
**Public healthcare insurance** [b]	3·47 (2·55 – 4·73)	3·53 (2·59-4·82)
**Migrant** [c]	3·00 (2·48 – 3·63)	4·00 (3·28-4·87)
ln (p) [d]		-0-44 (-0·37- -0·51)

Notes. TB: Tuberculosis. CI: Confidence Interval. N persons years: events (New-onset TB)/time of follow-up in years. Ref. Reference category. HR= Hazard ratios. ICU= Intensive care units. [a] At least one major chronic comorbidity (lung, kidney or liver disease) reported at the time of SARS-CoV-2 identification. [b] Belong to the Chilean public healthcare insurance. [c] Migrant status, regardless of legal residency, as recorded by the Chilean health system. [d] The natural logarithm of the Weibull shape parameter. All statistical tests were two-sided.

Supplementary figures (S1-S2)

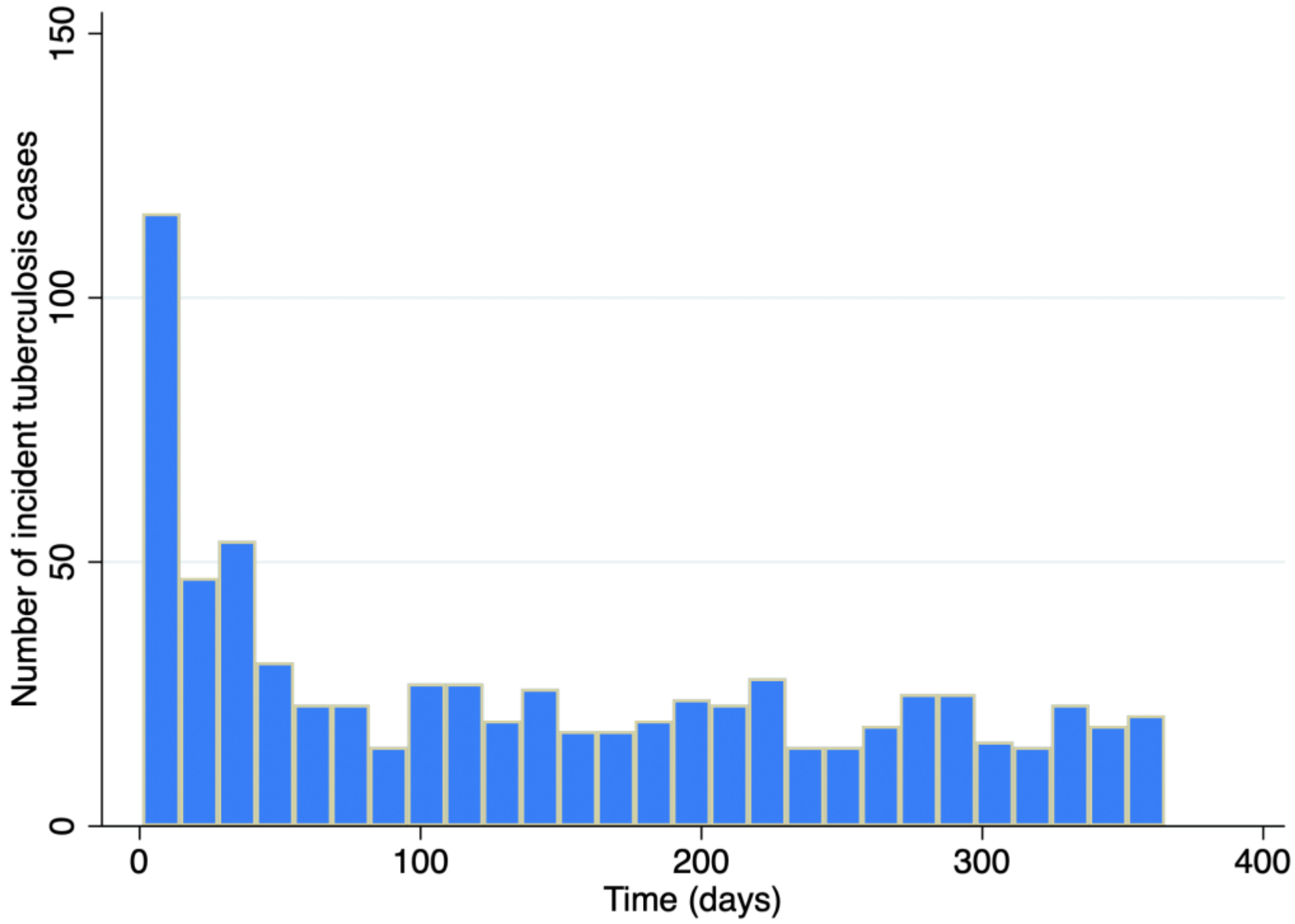

Figure S1. Distribution of time from SARS-CoV-2 infection diagnosis to new-onset tuberculosis within one year of follow-up (n = 733).

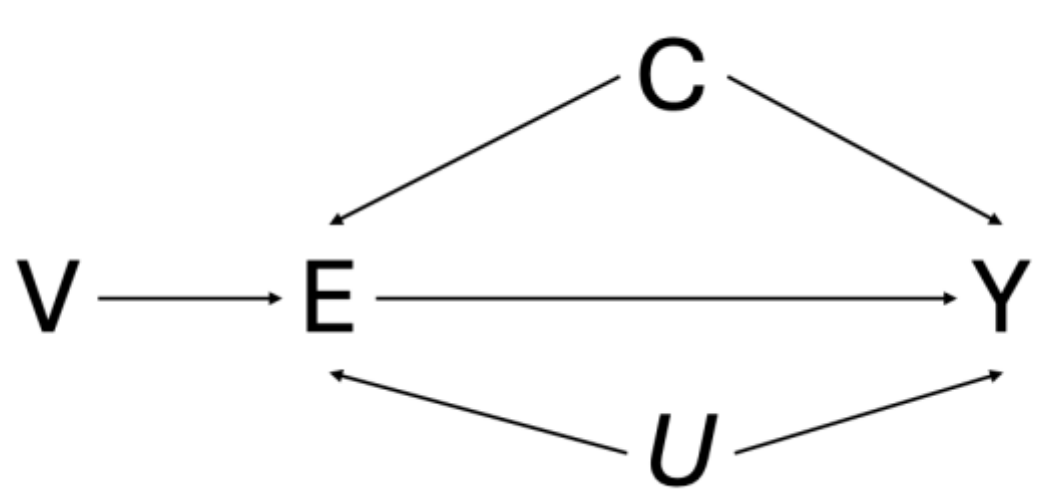

Figure S2. Directed acyclic graph (DAG) illustrating the hypothesized causal relationship between COVID-19 severity (*E*) and new-onset TB (*Y*). Measured confounders (*C [age, sex, migrant status, diabetes, comorbidities- lung, kidney or liver crhonic disease- and public healthcare insurance]*) and unmeasured confounders (U) are assumed to affect both exposure and outcome. SARS-CoV-2 vaccination status (*V*) is considered an effect modifier of the $E \rightarrow Y$ association and is represented as influencing only the exposure. Covariates included in the models were selected based on biological plausibility and clinical or epidemiological relevance, as guided by this DAG.[9]

References for the Supplementary Material

1. Taramasco, C., Rimassa, C., Romo, J.A., Césped Zavando, A. & Fuentes Bravo, R. Epidemiological surveillance in COVID-19 pandemic: EPIVIGILA system. *Medwave* 22, e2560 (2022).
2. Pan American Health Organization. COVID-19 vaccination country profiles. https://www.paho.org/es/temas/inmunizacion/datos-estadisticas-inmunizacion/perfiles-pais-vacunacion-covid-19 (2023).
3. Ministerio de Salud de Chile. Chile cumplió el 80% de su población vacunada contra SARS-CoV-2. https://www.minsal.cl/chile-cumplio-el-80-de-su-poblacion-vacunada-contra-sars-cov-2/ (2023).
4. Bitrán, R., Muñoz, J., Aguad, P., Navarrete, M. & Ubilla, G. Equity in the financing of social security for health in Chile. *Health Policy* 50, 171–196 (2000).
5. Aguilera, X., Castillo-Laborde, C., Ferrari, M.N., Delgado, I. & Ibáñez, C. Monitoring and evaluating progress towards universal health coverage in Chile. *PLoS Med* 11, e1001676 (2014).
6. Lozano, R. et al. Measuring universal health coverage based on an index of effective coverage of health services in 204 countries and territories, 1990–2019: a systematic analysis for the Global Burden of Disease Study 2019. *Lancet* 396, 1250–1284 (2020).
7. Centro de Epidemiología y Políticas de Salud, Facultad de Medicina Clínica Alemana–Universidad del Desarrollo. Estructura y funcionamiento del sistema de salud chileno, 2.ª edn (Universidad del Desarrollo, 2019).
8. Gallegos, E. & Muñoz, A. Protección financiera en salud: evolución y comparación del gasto de bolsillo de los hogares chilenos. *Rev Med Chile* 146, 737–744 (2018).
9. VanderWeele, T.J., Hernán, M.A. & Robins, J.M. Causal directed acyclic graphs and the direction of unmeasured confounding bias. *Epidemiology* 19, 720–728 (2008).
