# Supplementary material for: Risk of incident tuberculosis after severe COVID-19: a nationwide cohort study
Source: Nat Commun. 2026 Jun 29;17:8124. doi: 10.1038/s41467-026-74528-5 (PMC13458518; doi:10.1038/s41467-026-74528-5)
Supplement: Supplementary file 3 [file 41467_2026_74528_MOESM3_ESM.pdf]

nature portfolio

Peer Review File

Risk of incident tuberculosis after severe COVID-19: a nationwide cohort study

Corresponding Author: Dr María Elvira Balcells

This manuscript has been previously reviewed at another journal. This document only contains information relating to versions considered at Nature Communications.

This file contains all reviewer reports in order by version, followed by all author rebuttals in order by version.

Version 0:

Reviewer comments:

Reviewer #1

(Remarks to the Author)
General Comments
This is a well-designed study that evaluates the association between COVID-19 infection and its severity with TB disease risk with 1 yr follow-up.
The study design and analytical approaches are appropriate and thorough. This is a significant contribution to the study of COVID-19 and associated complications and the risk it confers for other conditions. The authors outline some potential clinical pathways through which TB disease risk is conferred.
Study design: Cohort study ; Population: Chile, Adults previously (within 1 year) diagnosed with SARS-Cov-2 infection; Data source: 5 national databases, national surveillance system
Outcome: new TB diagnosis; Exposure: COVID-19 severity and risk of developing Xpert-confirmed TB.; Covariates: socio-demographics and medical history; Effect modifier – COVID-19 vaccination; Analysis: time-to-event analysis
Data quality seems robust, the database systems uses unique ID numbers that enable follow-up and linkage of patient records. Population of relatively healthy individuals no immunocompromising conditions
The results indicate an increased risk of developing TB disease by CoVID-19 severity categories. There is attenuated risk with longer follow-up.
Specific Comments
1. Deaths during follow-up? Any competing-risk analysis was done as appropriate but should the authors not report conditional hazard ratios in that case?
2. Line 247,the authors should specify that this refers to median age "the onset TB were older (50 vs 40 years, p<0·001)"
3. A larger proportion of those who had covid vaccination developed TB, suggesting con-founding by co-morbidities or other medical history factors, if say a larger proportion of those with comorbidities vaccinated and also more vulnerable to TB disease?.
Further down in the results section, a stratified analysis shows increased risk among unvaccinated, suggesting effect modification.
4. Notably missing among the co-morbid conditions adjusted for in the analysis was cardiovascular disease. Please clarify the rationale for its exclusion and discuss any potential implications for the study findings.
5. The manuscript appears to assume that, following publication of the RECOVERY trial, patients with severe COVID-19 were treated with corticosteroids and immunomodulators. While this is understandable from a temporal and perhaps a guideline perspective, treatment uptake is likely to have been heterogeneous across settings and individuals. Please discuss whether inferring individual-level treatment exposure from a calendar period may introduce ecological fallacy or exposure misclassification, and clarify how this assumption may affect the interpretation of the findings.

Reviewer #2

(Remarks to the Author)
I co-reviewed this manuscript with one of the reviewers who provided the listed reports. This is part of the Nature Communications initiative to facilitate training in peer review and to provide appropriate recognition for Early Career Researchers who co-review manuscripts.

Reviewer #3

(Remarks to the Author)
Dear Authors

Thank you for submitting this interesting study. Please see below some comments

Introduction
1. Update TB numbers to those reported for 2024 from The Global TB Report 2025
2. Line 81-83. Approximately what percentage progress to these? Please add with reference

Methods
3. Can you comment on the completeness of these registries used for this study?
4. Given the exclusion for iv, is it possible someone admitted to ICU for respiratory distress and started on steroids while there PCR/antigen test is pending be excluded?
5. Is the exposure variable time-varying? Like if someone was non-hospitalized first and then hospitalized, how would they be categorized?
6. Was vaccination treated just as complete/non-complete or partial as well?
7. Did you account for multiple COVID infections? It is possible for someone to be reinfected and the second infection may result in a different exposure per your definitions.

Results
8. Figure 1. Can you please use a different color scheme. I was having a hard time differentiate different exposures.
9. Why is the aHR lower for ICU admitted patients as compared to hospitalized patients in the adjusted model? CIs are overlapping so no difference it seems.

Is this because that ICU admission (most severe COVID) leads to early development of TB so if we look at 6 months rather than 1 year, the dose response would be clearer?
You do make a comment about survivor bias in the discussion. Any other explanation for this.

Discussion
10. Line 398. I would suggest using the word viewpoint rather than criteria for Bradford-Hill. It is not a checklist but guidelines to determine causality.
https://pmc.ncbi.nlm.nih.gov/articles/PMC8206235/

Version 1:

Reviewer comments:

Reviewer #2

(Remarks to the Author)
I co-reviewed this manuscript with one of the reviewers who provided the listed reports. This is part of the Nature Communications initiative to facilitate training in peer review and to provide appropriate recognition for Early Career Researchers who co-review manuscripts.

Open Access This Peer Review File is licensed under a Creative Commons Attribution 4.0 International License, which permits use, sharing, adaptation, distribution and reproduction in any medium or format, as long as you give appropriate credit to the original author(s) and the source, provide a link to the Creative Commons license, and indicate if changes were made.
In cases where reviewers are anonymous, credit should be given to 'Anonymous Referee' and the source.
The images or other third party material in this Peer Review File are included in the article's Creative Commons license, unless indicated otherwise in a credit line to the material. If material is not included in the article's Creative Commons license and your intended use is not permitted by statutory regulation or exceeds the permitted use, you will need to obtain permission directly from the copyright holder.
To view a copy of this license, visit https://creativecommons.org/licenses/by/4.0/

Rebuttal letter, first revision

Risk of incident tuberculosis after severe COVID-19: a nationwide cohort study

We thank the reviewers for their comments and suggestions, which helped us improve our manuscript. We have addressed all concerns in a point-by-point response. The reviewers' comments are numbered consecutively, and our responses are labeled as "Response." References cited in our responses are listed at the end of this document. Page and line numbers refer to the clean version of the manuscript without tracked changes. Additionally, we have made editorial and formatting adjustments.

Comments

Reviewer #1 (Remarks to the Author):

1.1. Deaths during follow-up? Any competing-risk analysis was done as appropriate but should the authors not report conditional hazard ratios in that case?

Response: Thank you for making this observation.

The primary analyses estimated cause-specific (conditional) hazard ratios, quantifying the immediate risk of developing TB among individuals who are alive and TB-free at each time point. This modelling strategy was chosen to address the etiological question of whether severe COVID-19 is associated with an increased risk of tuberculosis, independent of the competing risk of death. Deaths due to COVID-19 and other causes were therefore treated as competing events. We now explicitly clarify that cause-specific HRs were our primary estimates, and that subdistribution HRs from Fine–Gray models were used to evaluate robustness of results. Specifically, we have clarified this in the Disussion section, (Page 10, lines 292-295) and Sensitivity and Robustness Analysis section (page 15, lines 430-432).

1.2. Line 247, the authors should specify that this refers to median age "the onset TB were older (50 vs 40 years, p<0·001)"

Response:

Thank you for the comment. We have revised the text on page 4, line 109, to explicitly indicate that the reported ages correspond to the median age.

1.3. A larger proportion of those who had covid vaccination developed TB, suggesting con-founding by co-morbidities or other medical history factors, if say a larger proportion of those with comorbidities vaccinated and also more vulnerable to TB disease? Further down in the results section, a stratified analysis shows increased risk among unvaccinated, suggesting effect modification.

Response:

We agree that the higher proportion of incident TB among vaccinated individuals in descriptive analyses probably reflects confounding by age and comorbidity burden, as vaccinated individuals were older and had a higher prevalence of chronic conditions associated with TB risk (Supplementary Table S1).

For this reason, COVID-19 vaccination status was included as an independent variable and explicitly examined as a potential effect modifier (first, by testing an interaction between COVID-19 severity and vaccination status, which was statistically significant; Supplementary Material Table S2). In stratified analyses, we observed that the association between COVID-19 severity and subsequent TB risk was stronger among unvaccinated individuals, despite this group being younger and having a lower burden of comorbidities (Table 2).

This pattern supports effect modification rather than residual confounding, because if confounding by age or comorbidity were driving the association, we would expect the vaccinated (older/comorbid) group to show the strongest association. Instead, we observed the opposite pattern, suggesting that the relationship between severe COVID-19 and later tuberculosis differs by vaccination status, which strengthens our findings by depicting that severe COVID-19 confers an increased TB risk even in populations with a lower baseline vulnerability.

We have clarified this point in the manuscript (page 7, lines 207–212) to avoid misinterpretation that SARS-CoV-2 vaccination directly reduces the risk of incident tuberculosis. Instead, vaccination appears to act as an upstream effect modifier by reducing the risk of severe COVID-19, which is strongly associated with subsequent TB risk.

1.4. Notably missing among the co-morbid conditions adjusted for in the analysis was cardiovascular disease. Please clarify the rationale for its exclusion and discuss any potential implications for the study findings.

Response:

We thank you for this comment. Covariate selection was guided by a directed acyclic graph (DAG) developed a priori (Supplementary Figure S2). Based on this causal framework, although cardiovascular disease is a well-established predictor of severe COVID-19[1], it is not independently associated with an increased risk of developing active TB.

To further assess the potential impact of this decision, we fitted a sensitivity model including cardiovascular disease as an independent variable to the covariates included in the main analysis (Table S5). The inclusion of this variable did not change the estimated associations (i.e., effect sizes or statistical significance), including the effect estimates for COVID-19 severity. Cardiovascular disease was not independently associated with TB risk in the adjusted model. Moreover, the model fit did not improve, as reflected by a higher Akaike Information Criterion (AIC) compared with the primary model ($AIC_{original}$: 15,045 vs AIC_{new_model}:15,047).

We also fitted a fully saturated model that included all available variables. Variables excluded from the final model lay outside the prespecified causal framework, and their inclusion did not improve model goodness-of-fit nor achieve statistical significance (Supplementary Table S6).

Taken together, these findings support excluding cardiovascular disease from the final model, as its inclusion does not change the results or improve model performance and is not required by the underlying causal structure.

To address the reviewer's concern and for clarity, we have now added this sensitivity analysis in the supplementary material. We have clarified this point in the Results section (Page 6, lines 181-183).

1.5. The manuscript appears to assume that, following publication of the RECOVERY trial, patients with severe COVID-19 were treated with corticosteroids and immunomodulators. While this is understandable from a temporal and perhaps a guideline perspective, treatment uptake is likely to have been heterogeneous across settings and individuals. Please discuss whether inferring individual-level treatment exposure from a calendar period may introduce ecological fallacy or exposure misclassification, and clarify how this assumption may affect the interpretation of the findings.

Response:

We agree with the reviewer that inferring individual-level corticosteroid or immunomodulator use from a calendar period may introduce ecological fallacy and

exposure misclassification (e.g., variation in treatment uptake between hospitals, clinical teams, and patients). To address this concern, we have revised the limitations section in the manuscript to explicitly acknowledge that post-RECOVERY stratified analyses are intended to assess robustness to temporal changes in the standard of care rather than to infer specific treatment effects.

We have updated the limitations section accordingly (page 9, lines 266-271)

Reviewer #3 (Remarks to the Author):

Thank you for submitting this interesting study. Please see below some comments

Introduction
3.1. Update TB numbers to those reported for 2024 from The Global TB Report 2025
3.2. Line 81-83. Approximately what percentage progress to these? Please add with reference

Response:

Thank you for your careful review of our manuscript.

First, we have updated the global tuberculosis burden estimates using the most recent data from the WHO Global Tuberculosis Report 2025 (Page 2, Lines 53-54).[2]

Second, we have revised page 2, lines 60-61 to specify the approximate proportion of SARS-CoV-2 infections that progress to severe or critical disease. An appropriate epidemiological reference has been added.[3]

Methods
3.3. Can you comment on the completeness of these registries used for this study?

Response:
We thank the reviewer for this important comment. All data sources used in this study are nationwide administrative and surveillance registries with near-complete population coverage across Chile, encompassing both public and private healthcare sectors. Records are deterministically linked using a unique personal identifier, enabling longitudinal follow-up while minimizing duplication and loss to follow-up.

Regarding data completeness, the outcome (incident TB) had no missing data, as individuals not recorded in the national TB registry during follow-up were classified as non-cases by the surveillance system's design. Similarly, the exposure variable (COVID-19 severity) had no missing data among individuals included in the COVID-19 registry.

Baseline covariates showed very low levels of missingness. Age, sex, and migrant status were cross-validated and supplemented using additional linked national databases, resulting in minimal residual missing data (e.g., missing sex data: n=1,433 of 3,607,175; 0.04%). Comorbidities were recorded using a predefined categorical registry system and subsequently operationalized as indicator (dummy) variables; therefore, no missingness was present in these variables within the analytical dataset.

Regression analyses were conducted using complete-case methods, as implemented by default in Stata. Given the extremely low proportion of missing data, no multiple imputation was performed. The minimal extent of missingness is unlikely to have materially influenced the estimates.

We have clarified this in the Statistical analysis section on page 15, lines 433-434. A comprehensive description of the data sources can also be found in the Supplementary Material (Dataset description, page 3).[4]

3.4. Given the exclusion for iv, is it possible someone admitted to ICU for respiratory distress and started on steroids while there PCR/antigen test is pending be excluded?

Response:
We thank the reviewer for raising this important point. The exclusion criterion (iv) referred specifically to pre-existing chronic immunocompromising conditions or long-term immunosuppressive therapy documented at the time of SARS-CoV-2 diagnosis. Immunosuppression status (yes/no) was determined using registry information available at the time of COVID-19 diagnosis and did not include treatments initiated subsequently for acute COVID-19 management.

Therefore, short-term corticosteroid therapy started during hospitalization or ICU admission for acute respiratory distress, including situations in which SARS-CoV-2 test results were pending, was not considered an exclusion criterion, as these treatments occurred after cohort entry and were part of the clinical management of COVID-19 rather than pre-existing immunosuppression.

We acknowledge that, in rare instances, a patient initially admitted with respiratory distress and treated empirically with corticosteroids might have subsequently tested positive for

SARS-CoV-2 and been recorded as immunosuppressed in administrative data. However, such scenarios would be exceptional and unlikely to introduce systematic bias, since immunosuppression status was defined based on pre-existing conditions documented at diagnosis.

Moreover, any potential misclassification arising from these situations would likely be non-differential with respect to the outcome and therefore would bias estimates toward the null rather than exaggerate the association between COVID-19 severity and incident tuberculosis.

We have clarified this definition in the Discussion (page 10, lines 281–285) and in the Methods section (page 12, lines 344–348).

3.5. Is the exposure variable time-varying? Like if someone was non-hospitalized first and then hospitalized, how would they be categorized?

Response:

The exposure variable (COVID-19 severity) was not modeled as time-varying. Instead, severity was hierarchically classified for each individual based on the most severe clinical presentation recorded during the index SARS-CoV-2 episode and was fixed at cohort entry.

Specifically, individuals were categorized by the highest level of care reached (non-hospitalized, hospitalized without ICU admission, or ICU admission), regardless of initial presentation. Therefore, individuals who were initially non-hospitalized but subsequently required hospitalization or ICU care during the same SARS-CoV-2 infection episode were classified according to the highest severity attained, irrespective of the sequence of events. This approach avoids misclassifying individuals whose clinical condition deteriorated after their initial diagnosis.

This strategy reflects the natural progression of COVID-19, in which severity evolves over time, while ensuring a clear temporal ordering between exposure classification and subsequent TB risk assessment. The hierarchical approach provides a consistent operational definition and avoids splitting follow-up time across multiple severity categories.

This point has been clarified in the Discussion section as a potential limitation (Page 10, lines 277-279). In addition we have updated the description of the exposure variable to improve transparency and understanding (Page 12, lines 350-352).

3.6. Was vaccination treated just as complete/non-complete or partial as well?

Response:

Vaccination status was treated as a binary variable (complete vs non-complete). Complete vaccination was defined according to Chilean national protocols at the time[5,6], accounting for vaccine-specific primary schedules (one dose for Ad5-nCoV or two doses for mRNA, inactivated, or viral vector vaccines).

Individuals who had received only one dose of a two-dose regimen at the time of SARS-CoV-2 infection were classified as non-completely vaccinated. We did not model partial vaccination or number of doses as separate categories, given the substantial heterogeneity in vaccination histories across the study period. In later phases of the pandemic, many individuals received different vaccine products for primary immunization and subsequent booster doses, resulting in diverse vaccination combinations prior to infection.

Because vaccination was evaluated as an effect modifier rather than a primary exposure, collapsing vaccination status into a binary variable improved interpretability, statistical stability, and adequate power within strata, while adequately addressing the study objective.

We have clarified this point in the Discussion section (page 10, lines 298-304) and in the Methods section (page 13, lines 377-383). In addition, we have also explicitly stated this definition in the table footnotes in the main text and the Supplementary Material (Tables 2 and 4, and Tables S1-S2).

3.7. Did you account for multiple COVID infections? It is possible for someone to be reinfected and the second infection may result in a different exposure per your definitions.

Response:

Thank you for this thoughtful comment. We did not account for multiple SARS-CoV-2 infections per individual. The cohort was defined based on the first documented SARS-CoV-2 infection (index infection) episode, and individuals with subsequent infection records were not reclassified or re-entered with a new exposure status.

This design choice was intentional. Multiple infections could further alter immune function and increase susceptibility to tuberculosis independently of COVID-19 severity, thereby introducing additional heterogeneity and potentially biasing estimates away from the null.[7] By restricting the analysis to the index infection, we aimed to preserve a clear

temporal structure, internal validity, and provide a clearer assessment of the association between COVID-19 severity and subsequent TB risk.

The potential cumulative or dose–response effect of repeated SARS-CoV-2 infections on TB risk remains an important question for future research, but was beyond the scope of the present study.

We have clarified this point in the Discussion (page 10, lines 279–281), and in the Methods section (study population; page 11, line 336–340 & page 12, lines 337-338).

Results
3.8. Figure 2. Can you please use a different color scheme. I was having a hard time differentiate different exposures.

Response:
Thank you for the suggestion. We restructured the figure by incorporating greater transparency to improve visual discrimination across strata, enhancing the clarity of the probability of remaining TB-free and its corresponding confidence intervals. (Figure 2).

3.9. Why is the aHR lower for ICU admitted patients as compared to hospitalized patients in the adjusted model? CIs are overlapping so no difference it seems. Is this because that ICU admission (most severe COVID) leads to early development of TB so if we look at 6 months rather than 1 year, the dose response would be clearer? You do make a comment about survivor bias in the discussion. Any other explanation for this.

Response:
We agree with the reviewer that the overlapping confidence intervals indicate no statistically significant difference between hospitalized and ICU-admitted patients in the fully adjusted 1-year models. First, as discussed in the manuscript, our interpretation emphasizes temporal risk patterns rather than single-point estimates.

Although our three categories could have been collapsed into just non-hospitalized versus hospitalized (including ward and ICU admissions), we deliberately retained a separate ICU-admitted stratum to illustrate the potential impact of survivor bias. Individuals requiring ICU care likely experienced the highest biological susceptibility to TB due to profound immune dysregulation; however, this increased risk may be underestimated because many did not survive long enough to develop the outcome. Despite this attenuation, ICU survivors remain a clinically important subgroup, warranting particular

attention both during the critical illness phase and after hospital discharge among those who survive.

Second, when restricting follow-up to the first 6 months after a COVID-19 pneumonia the TB risk is even higher. This pattern is biologically and clinically plausible and likely reflects a combination of short-term immune dysregulation following severe COVID-19 and intensified clinical surveillance among ICU-admitted patients, which may facilitate earlier TB detection during the acute and post-acute SARS-CoV-2 infection.

Beyond the early post-infection window, the association attenuates (Results section, page 6, lines 152-156 & page 6, 157-160), and the increasing influence of survivor and competing risks, which are addressed in the Discussion (Page 8, lines 218-228). Taken together, these findings support the interpretation that the excess TB risk following severe COVID-19 is concentrated in the early months after infection rather than uniformly sustained over longer follow-up.

We have now added the early post-infection analysis (first six months) to Table 3, to more clearly show this early risk.

3.10. Line 398. I would suggest using the word viewpoint rather than criteria for Bradford-Hill. It is not a checklist but guidelines to determine causality. **https://pmc.ncbi.nlm.nih.gov/articles/PMC8206235/**

Response:

We thank the reviewer for this helpful suggestion. We agree that the Bradford Hill framework should be referred to as viewpoints rather than criteria, as it is not intended to function as a checklist for causal inference. We have revised the text accordingly; the changes can be found on page 9, line 256.

Thank you for your time and careful review.

References

1. Russell CD, Lone NI, Baillie JK. Comorbidities, multimorbidity and COVID-19. *Nat Med*. *Nature Research*. 2023;29(2):334-343. doi:10.1038/s41591-022-02156-9
2. World Health Organization (WHO). *2025 Tuberculosis Global Report*. 2025.
3. Fu L, Wang B, Yuan T, et al. Clinical characteristics of coronavirus disease 2019 (COVID-19) in China: A systematic review and meta-analysis. *Journal of Infection*. 2020;80(6):656-665. doi:10.1016/j.jinf.2020.03.041

4. Taramasco C, Rimassa C, Romo JA, Césped Zavando A, Fuentes Bravo R. Epidemiological surveillance in COVID-19 pandemic: EPIVIGILA system. *Medwave*. *Medwave Estudios Ltda*. 2022;22(5). doi:10.5867/medwave.2022.05.2560
5. Aguilera X, González C, Apablaza M, et al. Immunization and SARS-CoV-2 Antibody Seroprevalence in a Country with High Vaccination Coverage: Lessons from Chile. *Vaccines (Basel)*. 2022;10(7). doi:10.3390/vaccines10071002
6. Jara A, Undurraga EA, González C, et al. Effectiveness of an Inactivated SARS-CoV-2 Vaccine in Chile. *New England Journal of Medicine*. 2021;385(10):875-884. doi:10.1056/nejmoa2107715
7. Bowe B, Xie Y, Al-Aly Z. Acute and postacute sequelae associated with SARS-CoV-2 reinfection. *Nat Med*. 2022;28(11):2398-2405. doi:10.1038/s41591-022-02051-3
